# Specifications for Modelling of the Phenomenon of Compression of Closed-Cell Aluminium Foams with Neural Networks

**DOI:** 10.3390/ma15031262

**Published:** 2022-02-08

**Authors:** Anna M. Stręk, Marek Dudzik, Tomasz Machniewicz

**Affiliations:** 1 Faculty of Civil Engineering, Cracow University of Technology, 31-155 Cracow, Poland; 2 Faculty of Electrical and Computer Engineering, Cracow University of Technology, 31-155 Cracow, Poland; marekdudzik@pk.edu.pl; 3 Faculty of Mechanical Engineering and Robotics, AGH University of Science and Technology, 30-059 Cracow, Poland; machniew@agh.edu.pl

**Keywords:** artificial neural network design, two-layered feedforward network, compressive behaviour, aluminium foam

## Abstract

The article presents a novel application of the most up-to-date computational approach, i.e., artificial intelligence, to the problem of the compression of closed-cell aluminium. The objective of the research was to investigate whether the phenomenon can be described by neural networks and to determine the details of the network architecture so that the assumed criteria of accuracy, ability to prognose and repeatability would be complied. The methodology consisted of the following stages: experimental compression of foam specimens, choice of machine learning parameters, implementation of an algorithm for building different structures of artificial neural networks (ANNs), a two-step verification of the quality of built models and finally the choice of the most appropriate ones. The studied ANNs were two-layer feedforward networks with varying neuron numbers in the hidden layer. The following measures of evaluation were assumed: mean square error (*MSE*), sum of absolute errors (*SAE*) and mean absolute relative error (*MARE*). Obtained results show that networks trained with the assumed learning parameters which had 4 to 11 neurons in the hidden layer were appropriate for modelling and prognosing the compression of closed-cell aluminium in the assumed domains; however, they fulfilled accuracy and repeatability conditions differently. The network with six neurons in the hidden layer provided the best accuracy of prognosis at MARE≤2.7% but little robustness. On the other hand, the structure with a complexity of 11 neurons gave a similar high-quality of prognosis at MARE≤3.0% but with a much better robustness indication (80%). The results also allowed the determination of the minimum threshold of the accuracy of prognosis: MARE≥1.66%. In conclusion, the research shows that the phenomenon of the compression of aluminium foam is able to be described by neural networks within the frames of made assumptions and allowed for the determination of detailed specifications of structure and learning parameters for building models with good-quality accuracy and robustness.

## 1. Introduction

### 1.1. Problem Origins

Closed-cell aluminium is a well-known engineering material, mostly used where light-weight applications require satisfactory mechanical properties [1,2,3] or energy absorption as a determinant [2,4]. Other properties, which make this material multifunctional, are: sound wave attenuation [5,6], electromagnetic wave absorption [7,8], vibration intimidation [9], thermal conductivity [10,11], relatively easy shape tailoring [12] and potential for usage in composites [13,14,15]. Examples of the usage of aluminium foams include, among others: the automotive industry, space industry, energy and battery field, military applications and machine construction [16,17,18,19,20]. We would also like to highlight civil engineering and architecture here, since these application fields are unjustly underestimated in the metal foam industry even though they have significant potential. Examples of the usage of closed and open cellular metals include: structural elements (e.g., wall slabs, staircase slabs, parking slabs) [17,21,22], interior and exterior architectural design [23,24], highway sound absorbers [5,25], architectural electromagnetic shielding [26], sound absorbers in metro tunnels [17], dividing wall slabs with sound insulation (e.g., for lecture halls) [27] and the novel concept of earthquake protection against building pounding [28].

Taking into consideration so much engineering and design interest in closed-cell aluminium foams, it is a natural consequence that much scientific attention is drawn to the appropriate description of this material in various aspects. A significant number of works focus on structural characterization, e.g., [16,29,30,31], property analysis, e.g., [1,2,3,4,5,6,7,8,9,10,11,32], manufacturing methods, e.g., [12,16,33], experimental investigations, e.g., [34,35,36,37,38,39] and modelling. As for modelling, this field is widely researched, and the number of publications about this subject is extensive. They cover the modelling of basic mechanical properties or constitutive relations with different approaches: the analytical derivation of models based on the foam’s cell geometry, incorporation of probabilistic approach, application of theory of elasticity and numerical solutions with finite-element methods, e.g., [40,41,42,43,44,45,46,47,48]. Application of the most up-to-date numerical tool, i.e., neural networks, to the modelling of mechanical characteristics of metal foams (open-cell) can be found in papers [49,50,51,52]. The authors are not aware of any works which apply this valuable method to the modelling of base relations in closed-cell metal foams. However, the authors note that neural networks have been used in the modelling of closed-cell polymer cellulars [53]. Neural networks are more often used for the analysis of specific features of metal foams and sponges, mainly heat exchange, e.g., [54,55].

### 1.2. Problem Statement and Proposed Solution’s Generals

There are extensive specific material models for closed-cell aluminium foams, which could be a starting point in the present discussion, such as the general relation given in Expression (1) [40]. It reflects the intuitive dependence of the mechanical behaviour of foam on its structural nature:

(1) $$\frac{\mathrm{cellular}\;\mathrm{material}\;\mathrm{property}}{\mathrm{skeleton}\;\mathrm{property}}=C{\left(\frac{\rho }{\rho_{s}}\right)}^{n},$$

Formula (1) relates by a power law a chosen cellular material’s property and a respective skeleton’s property to both the cellular material’s density ρ and skeleton’s density $\rho_{s}$. Parameters *C* and *n* are supposed to be determined experimentally for the given material. This formula may assume specific forms, depending on what kind of property is desired (compressive strength, material’s modulus, etc.) and on a general characterization of the considered material (closed- or open-cellular; elastic, plastic or brittle). However, it does not express a continuous model. Parameters *C* and *n* were already determined for some specific foams and sponges [40], but they are given mostly in the form of intervals of values and should be confronted with experimental data each time. Additionally, Relation (1) requires one to know the skeleton’s density and the respective skeleton’s property in order to determine the analogous foam’s property. This fact may be a serious inconvenience in the case when the foam is bought as a ready product from an external supplier and data about the skeleton’s material are inaccessible. However, despite all its shortcomings, the crucial premise of (1) is that a material’s density is reflected in its behaviour. This dependency was a key for the assumption of the form of a general relationship that was the basis for neural network modelling in the presented research:

(2) σ=f(ε,ρ).

Formula (2) refers to a general relationship between strain ε, the material’s apparent density ρ and its response in uniaxial static compression as expressed by stress σ.

The main goal of the presented research was then to use neural networks to find the most appropriate model, which, according to the above general formula, would be able to estimate a stress response for a given strain of a closed-cell aluminium foam of a given density.

Together with the assumption about the general form of Relation (2), some choices about the artificial intelligence tool also had to be determined. The discussion on neural network structural specifications such as the number of layers, activation functions, the number of inputs, the optimization of weights, the number of neurons, preprocessing of inputs, the choice of learning parameters, inclusion of statistical approach, etc., has been ongoing in recent years, e.g., [56,57,58,59,60]. However, it is a common belief that no universal method for assuming these parameters exists or that there is rather little guidance [57,61,62]. In consequence, the approach to each data set and each application has to be designed individually or within a class of similarity. In the face of a lack of prior works using neural networks for the modelling of closed-cell foams, it was decided that the main directions for the network structure in the presented study would be based on previous research on open-cell aluminium [49,50,51].

### 1.3. Research Significance

Artificial intelligence is an interesting, modern approach in engineering [63,64,65]. It can be used to address, among other things, mechanical problems in structural engineering [66,67], in civil engineering and architecture [68,69,70,71] and in material engineering [72,73,74,75,76]. As has already been said, metal foams can find their place in these fields, so building good-quality models for cellular metals with the help of neural networks is a new, tempting solution worth investigation and development. The starting point has already been reached for open-cell aluminium [49,50,51], and now the research has been continued for closed-cell aluminium foam—the results of which are reported in this article.

The presented research consisted of a few stages. It was decided compressive tests would first be performed to obtain experimental data for network training (Section 2 and Section 3.1). Next, a general form of the network structure was accepted: a two-layer feedforward network with a Levenberg–Marquardt training algorithm (Section 3.2). Specification of hyperparameters was performed in a specially designed algorithm (Section 3.3 and Section 3.4). Results were assessed in a two-step evaluation procedure according to assumed measures (Section 3.5 and Section 4). All in all, the research was aimed at answering the following questions:

- Is it possible to describe the phenomenon of compression of aluminium foams with a model generated from neural networks based on the assumed general relation?
- What assumptions/general choices about the networks’ structure and learning parameters should be determined?
- How should the obtained results be evaluated? What criteria and what measures should be assumed?
- What structure and learning parameters should be assumed to most adequately describe the phenomenon?
- Is the model valid only for the training data (particular model), or is it capable of prognosing for new data (general model)?

The obtained results prove that these questions can be answered positively and with details that are described in the present paper.

## 2. Material and Experiment

### 2.1. Material

**Figure 1:**
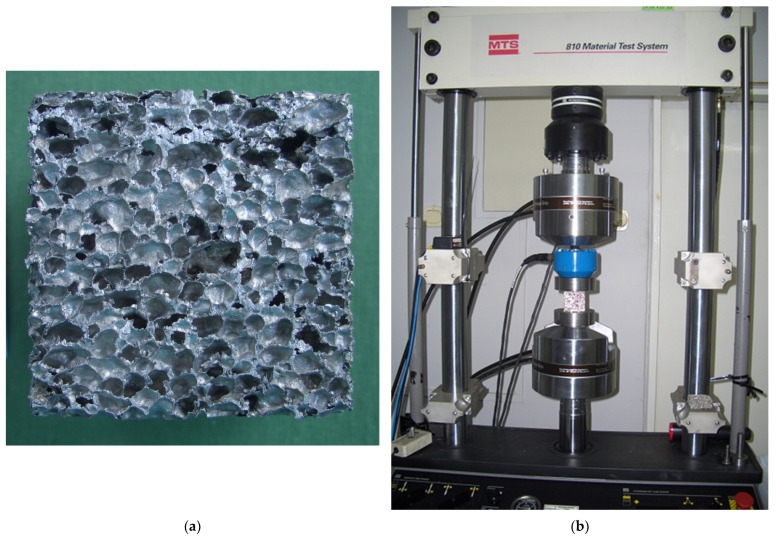
Experiment of uniaxial compression: (**a**) an aluminium foam cubic specimen; (**b**) experimental set with one of the samples between the presses ready for the compression test.

The studied material was aluminium foam with the following general morphological characteristics: closed-cell, stochastic and isotropic (in representative volume). Material was cut into cubic specimens of 5 × 5 × 5 cm^3^. Detailed samples’ dimensions were determined according to procedure from [77] with callipers with a 0.01 mm scale VIS (VIS, Warszawa, Poland) and Vogel 202040.3 (Vogel Germany GmbH & Co. KG, Kevelaer, Germany). Masses of foams were measured using balance WPS600/C (Radwag, Poland). Apparent density of specimens was calculated as the ratio of mass over volume; average density was ρ=0.240 g/cm^3^. Details of specimens’ characteristics are presented in Table 1, and a photo of one of the samples is shown in Figure 1a. Photos of all specimens before the experiments are enclosed in Appendix A.

### 2.2. Uniaxial Compression Experiments

Samples were tested using MTS 810 testing machine of class 1 (MTS Systems Corporation, Eden Prairie, MN, USA) with the additional force sensor interface (capacity: 25 kN). Experimental data were gathered with the help of the computer programme TestWorks4 (MTS Systems Corporation, Eden Prairie, MN, USA, version V4.08D). Photographs were taken with Casio Exilim EX-Z55 camera (Casio Computer Co, Ltd., Tokyo, Japan). The tests were conducted at room temperature. The compression procedure was performed quasi-statically—the strain rate was assumed as 2.5 × 10^−4^ m/s. The initial force (preload force) was assumed as 10 N. Figure 1b presents one of the specimens in the testing machine. All specimens were compressed up to strain ~70%. Testing was performed in accordance with the procedure from [34,35,36,37].

**Figure 2:**
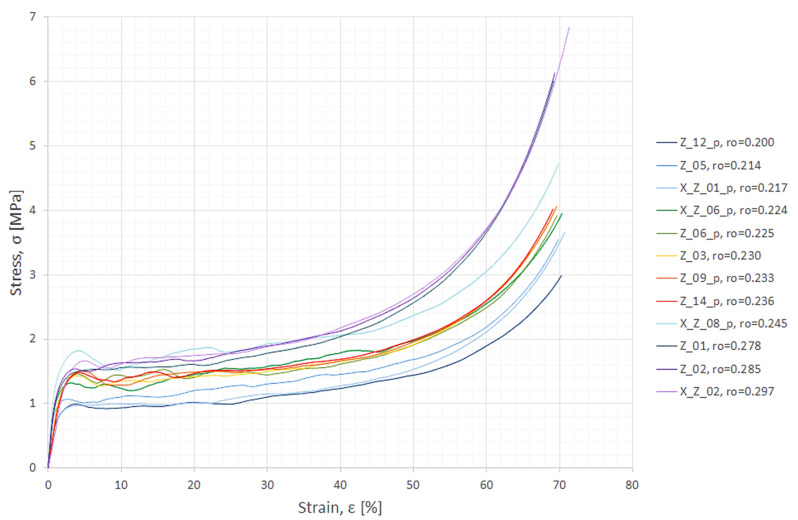
Stress–strain relationships from compression of aluminium foam specimens. Following the sample’s name is its density given in [g/cm^3^].

Figure 2 shows results of the compression experiments in the form of a stress–strain graph. One can observe that the material’s response is connected to its density so that the plot values of the lightest samples are the lowest and those of the heaviest are the highest. Additionally, all plots exhibit traits characteristic of compression of a closed-cell metal foam [40]: the initial steep region interpreted as the elastic phase, then the first local maxima associated with compressive strength, followed by the plateau region where densification occurs and lastly, a section where the curves become steep again. It is worth mentioning that during densification individual cells or cell groups collapse plastically, which is reflected in the graph by many local maxima and minima appearing alternately among the plateaus.

General material features, which were determined based on experimental results, included average compressive strength $\sigma_{c}=1.40$ MPa, average plateau strength $\sigma_{\mathrm{pl}}=1.44$ MPa and average plateau end $\varepsilon_{\mathrm{pl}.f}=45.78$%.

## 3. Methods: Computations with Artificial Neural Networks

The main concept of the research stage devoted to neural networks was to generate and train a considerable set of comparable networks, then assess them according to assumed criteria and finally—based on the choice of the ‘best’ network—determine the most adequate neural network structure and learning parameters for building a model of the phenomenon of closed-cell aluminium compression.

Before the realization of this idea, a set of assumptions was determined. The most important pre-choice was designing a two-step evaluation—this assumption affected all specific research actions. It was decided that we would generate and train all networks using experimental data for 11 (out of 12) specimens. Obtained networks were then evaluated for the first time in terms of whether they were good-quality models of compression of those particular 11 samples. This step was important from the point of view of understanding the complexity of the physical phenomenon of aluminium foam compression, which was approached to be described. The data for the left-out sample (12th) were used in the second evaluation in terms of whether the networks were capable of adequate prognosis. This step was important for the generalization potential of the obtained models.

Other assumptions involved choosing neural network learning parameters, designing the path for the building and training of networks and selection of criteria measures. They will be discussed below, together with the detailed description of the proposed computational method. First, the processing and preparation of experimental data will be reported (Section 3.1). Next, detailed information about the structure of the assumed networks will be given (Section 3.2). Following that, the choice of learning parameters (Section 3.3.) and the algorithm used to build and train networks (Section 3.4) will be described. Finally, evaluation criteria for accuracy will be discussed (Section 3.5).

Calculations were performed using Matlab R2017b and R2019A in conjunction with Excel 2016.

### 3.1. Data for the Networks

During the experimental stage 12 aluminium foam specimens underwent compression. Collected data were initially preprocessed to suit as arguments and targets for neural networks (Section 3.1.1). Thereafter, the data set was divided into parts dedicated to network learning and verification (Section 3.1.2). The last aspect of data preprocessing was normalization, and this had already been performed within the NN computations. The reverse procedure (denormalization) had to be performed after the training of networks (Section 3.1.2).

#### 3.1.1. Initial Preprocessing of Experimental Data

**Figure 3:**
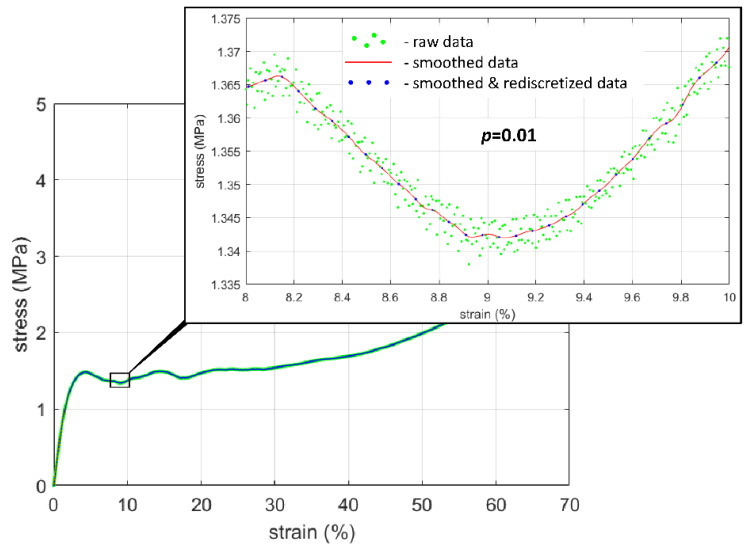
Exemplary comparison of raw stress–strain data with data smoothed using cubic smoothing splines with the smoothing parameter *p* = 0.01 (specimen Z_14_p).

**Figure A2:**
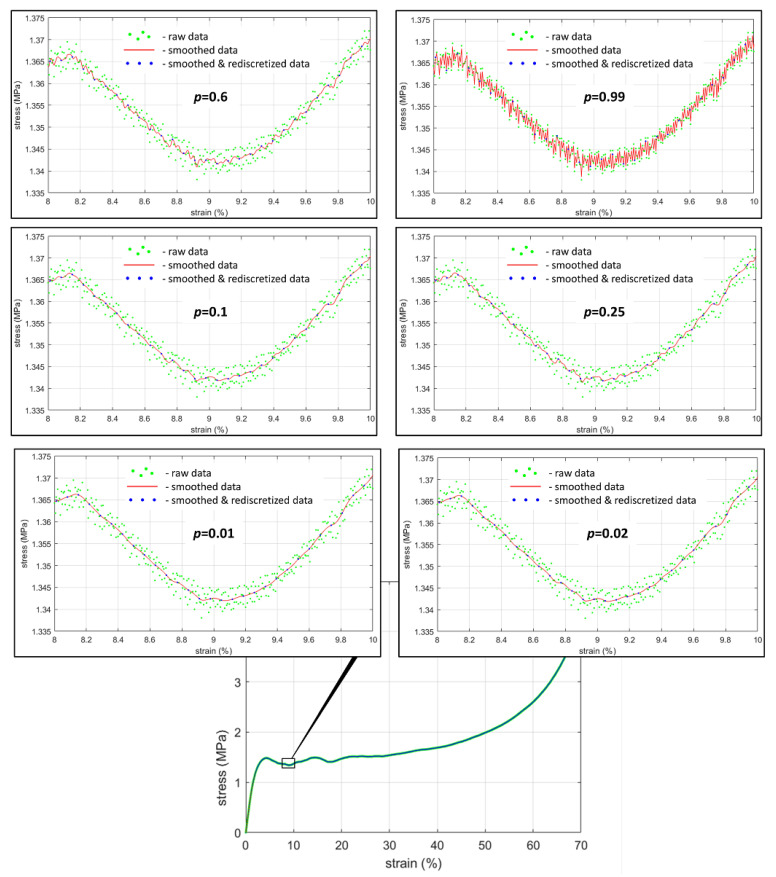
Comparison of the quality of smoothing of experimental data for exemplary values of parameter *p* (specimen Z_14_p).

Raw data collected during experiments with data acquisition frequency of 100 Hz were subject to initial preprocessing consisting of smoothing—to attenuate noise on the load and displacemnet transducer signals—and rediscretization—to set a uniform strain data vector, common for all specimens. Smoothing of the data was performed in the time domain using cubic smoothing splines [78,79]. The aim of smoothing was to eliminate the scatter of the raw data and, at the same time, to preserve the original stress–strain response, as exemplified in Figure 3. For this purpose the inbuilt Matlab function *csaps* was used with a smoothing parameter *p* = 0.01 [80]. The parameter’s *p* value was chosen by a visual examination of the stress–strain plots corresponding to the raw and smoothed data for the interval p∈〈0.01;0.99〉 (examples are depicted in Figure A2 in Appendix B). As a result, smoothed sigma–epsilon data for every specimen contained 1000 data pairs, in the strain range from 0 to 69%, which was the widest common range recorded for all specimens. Such a number of points ensured the possibility of sufficiently precise mapping of the considered stress–strain curves. Due to the assumption that the strain vector was common to all specimens, neural networks were expected to correctly predict only the stress values as the responses to the given strains. Simultaneously, the same number of data points assures the same impact of each specimen on the learning/validation process of neural networks.

Depending on specimens’ density and stochastic cellular structure their compressive response varied, which—let it be recalled again—can be seen in the above Figure 2: Plots for lighter samples are in the bottom part, while plots for the heavier ones are in the top. Density then, along with strain, had to be the input arguments for the networks. The target was to be stress. This is in agreement with the already cited theoretical approach as in Formula (1) and with the primary assumption expressed in Formula (2). Thus, arguments for the neural networks were set into *n* = 12,000 vectors (1000 for each sample): $A_{i}={\left[\varepsilon_{i},\rho_{i}\right]}^{T}$, where: i=1,2,…,n; $\varepsilon_{i}$—the initially preprocessed value of strain from experiments for the given sample in (%); and $\rho_{i}$—the apparent density of the given sample in (g/cm^3^). The targets were experimental values of stress $\sigma_{i}$ in (MPa) after initial preprocessing. The sequence of indices *i* in arguments and targets is of course corresponding.

#### 3.1.2. Division of the Data Set

The experimental data set obtained for 12 specimens was divided in general into two subsets:

1. Data of 11 specimens, which were devoted to building the NN model of the phenomenon of compression of these particular aluminium foam samples;
2. Data of 1 specimen, which were to be used later for verification of whether the obtained model could be used as a general model, that is, for prognosing the phenomenon of the compression of aluminium foam with respect to different materials’ apparent density.

As for building the particular model, the assumed neural network learning procedure consisted of three stages: training, validation (network self-verification) and testing (exposition to new data) [81,82,83]. So the data set from the compression of 11 specimens had to be subdivided to assure data for all three stages. It was assumed that 60% of the data would be devoted to the training phase, 20% to validation and 20% to testing. This was practically conducted by assigning sequentially every fifth input–target pair starting from *i* = 4 to the validation data subset and every fifth input–target pair starting from *i* = 5 to the test data subset. The remaining data constituted the data subset for the training phase. The division into subsets was not changed, so the subsets contained exactly the same data for each studied network.

Experimental results for specimen Z_14_p were separated as the data for verification of the prognosis capability. This sample was chosen because its graph was more or less in the middle of all individual stress–strain plots (Figure 2).

#### 3.1.3. Normalization and Denormalization

As for the normalization, the inbuilt Matlab function mapminmax was used [84,85,86]. This function is a linear transformation of data comprising a certain range into the interval of given desired boundaries and can be expressed as in Formula (3):

(3) $$V^{\prime }=\frac{V-V_{\min}}{V_{\max}-V_{\min}}\cdot \left({V^{\prime }}_{\max}-{V^{\prime }}_{\min}\right)+{V^{\prime }}_{\min}.$$

In Formula (3): V is the value to be transformed; $V^{\prime }$ is the new value; $V_{\max}$,$\;V_{\min}$ are original interval boundaries; and ${V^{\prime }}_{\max}$,$\;{V^{\prime }}_{\min}$ are the desired range boundaries (in normalization they are assumed as −1 and 1). In our study vectors $A_{i}$ were normalized respectively into the following input vectors: $X_{i}={\left[x_{1,i}=\varepsilon_{\mathrm{norm},i},\;x_{2,i}=\rho_{\mathrm{norm},i}\right]}^{T}$, where i=1,2,…,n.

Such prepared data were used in network training. Networks’ outputs, which were supposed to correspond to stresses, were obtained. Yet, their values were within the interval of normalization 〈−1,1〉: $y_{i}=\sigma_{\mathrm{appr}.\mathrm{norm},i}$. Hence, the reverse procedure of output denormalization was necessary: $y_{i}=\sigma_{\mathrm{appr}.\mathrm{norm},i}\overset{\mathrm{post}-\mathrm{processing}}{\to }y_{i}=\sigma_{\mathrm{appr},i}$.

### 3.2. Assumed Artifitial Networks Architecture

The authors assumed the general network structure type and activation function types according to what is recommended for nonlinear function approximation in the literature [87] and also to what had been proved to work well in a previously investigated case of open-cell metals [49,50].

**Figure 4:**
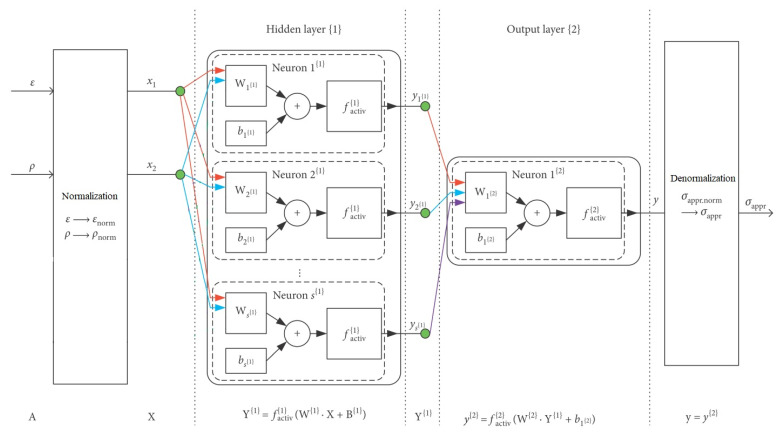
The structure of neural networks used in the study. All symbols are explained in the text (Section 3.2).

Figure 4 shows a detailed scheme of the network architecture, which will be explained below in detail. The index i=1, 2, …, n, which indicates the numbering of the given input data and the respective target, is omitted in the below discussion and Expressions (4)–(13) for simplification. This does not affect the logic of the reasoning since networks use all inputs and targets for training and verification, so all data (all *i*-s) are used, and each is only used once.

The neural network architecture was chosen as a feedforward network with two layers: one hidden layer, labelled in the research with {1} and one output layer labelled with {2}. Argument **A**, after normalization, entered the hidden layer {1} as input **X**. The number of neurons in the hidden layer was assumed as varying within the range $s^{\left\{1\right\}}=\langle 1;50\rangle$. The function tansig—a hyperbolic tangent sigmoid (mathematically equivalent to tanh [84])—was chosen as the activation function for the hidden layer. It was denoted as $f^{\left\{1\right\}}{}_{\mathrm{activ}}$ and expressed as in Formula (4):

(4) $$f^{\left\{1\right\}}{}_{\mathrm{activ}}\left(arg^{\left\{1\right\}}\right)=\frac{e^{2\cdot arg^{\left\{1\right\}}}-1}{e^{2\cdot arg^{\left\{1\right\}}}+1}=\tan h\left(arg^{\left\{1\right\}}\right),$$

where the argument of the transfer function in the hidden layer was defined as:

(5) $$arg^{\left\{1\right\}}=W^{\left\{1\right\}}\cdot X+B^{\left\{1\right\}}.$$

Symbols in Formula (5) denoted the following magnitudes:

- X—the input vector, mathematically formulated as in Equation (6) below;
- $B^{\left\{1\right\}}$—the column vector of biases for layer {1}, mathematically formulated as in Equation (7) below;
- $W^{\left\{1\right\}}$—the matrix of weights of inputs for layer {1}, mathematically formulated as in Equation (8) below:
  (6) $$X={\left[x_{1},\;x_{2}\right]}^{T},$$
  (7) $$B^{\left\{1\right\}}={\left[b_{1}^{\left\{1\right\}},b_{2}^{\left\{1\right\}},\;\ldots ,b_{p-1}^{\left\{1\right\}},\;b_{p}^{\left\{1\right\}},b_{p+1}^{\left\{1\right\}},\;\ldots ,\;b_{s^{\left\{1\right\}}}^{\left\{1\right\}}\right]}^{T},$$
  (8) $$W^{\left\{1\right\}}=\left[\begin{array}{cccc} w_{1,1}^{\left\{1\right\}} & w_{1,2}^{\left\{1\right\}} & w_{1,3}^{\left\{1\right\}} & w_{1,4}^{\left\{1\right\}}\\ w_{2,1}^{\left\{1\right\}} & w_{2,2}^{\left\{1\right\}} & w_{2,3}^{\left\{1\right\}} & w_{2,4}^{\left\{1\right\}}\\ \vdots & \vdots & \vdots & \vdots \\ w_{p-1,1}^{\left\{1\right\}} & w_{p-1,2}^{\left\{1\right\}} & w_{p-1,3}^{\left\{1\right\}} & w_{p-1,4}^{\left\{1\right\}}\\ w_{p,1}^{\left\{1\right\}} & w_{p,2}^{\left\{1\right\}} & w_{p,3}^{\left\{1\right\}} & w_{p,4}^{\left\{1\right\}}\\ w_{p+1,1}^{\left\{1\right\}} & w_{p+1,2}^{\left\{1\right\}} & w_{p+1,3}^{\left\{1\right\}} & w_{p+1,4}^{\left\{1\right\}}\\ \vdots & \vdots & \vdots & \vdots \\ w_{s^{\left\{1\right\}},1}^{\left\{1\right\}} & w_{s^{\left\{1\right\}},2}^{\left\{1\right\}} & w_{s^{\left\{1\right\}},3}^{\left\{1\right\}} & w_{s^{\left\{1\right\}},4}^{\left\{1\right\}} \end{array}\right]$$

Computations in the hidden layer {1} led to the column vector of outputs $Y^{\left\{1\right\}}$ of the hidden layer {1}. This vector had the form shown in Formula (9):

(9) $$Y^{\left\{1\right\}}={\left[y_{1}^{\left\{1\right\}},y_{2}^{\left\{1\right\}},\;\ldots ,y_{p-1}^{\left\{1\right\}},\;y_{p}^{\left\{1\right\}},y_{p+1}^{\left\{1\right\}},\;\ldots ,\;y_{s^{\left\{1\right\}}}^{\left\{1\right\}}\right]}^{T}.$$

Vector $Y^{\left\{1\right\}}$ then entered the output layer {2}. The number of neurons in layer {2} was unchangeable and was assumed as $s^{\left\{2\right\}}=1$, taking into account the single variable output [88]. The activation function for the output layer, $f^{\left\{2\right\}}{}_{\mathrm{activ}}$, was chosen as purelin [84] and expressed as in Formula (10):

(10) $$f^{\left\{2\right\}}{}_{\mathrm{activ}}\left(arg^{\left\{2\right\}}\right)=a\cdot arg^{\left\{2\right\}},$$

where a was a directional coefficient assumed as constant a=1 and the argument of the transfer function in the output layer was defined by the following Formula (11):

(11) $$arg^{\left\{2\right\}}=W^{\left\{2\right\}}\cdot Y^{\left\{1\right\}}+b_{1}^{\left\{2\right\}}.$$

Symbols in the above expression denote the following magnitudes:

- $Y^{\left\{1\right\}}$—the hidden layer outputs, as in Formula (9);
- $b_{1}^{\left\{2\right\}}$—the bias for the output layer, a scalar value;
- $W^{\left\{2\right\}}$—the row vector of weights of inputs for layer {2}, mathematically formulated as in Equation (12) below:
  (12) $$W^{\left\{2\right\}}=\left[\begin{array}{cccccccc} w_{1,1}^{\left\{2\right\}} & w_{1,2}^{\left\{2\right\}} & \ldots & w_{1,j-1}^{\left\{2\right\}} & w_{1,j}^{\left\{2\right\}} & w_{1,j+1}^{\left\{2\right\}} & \ldots & w_{1,s^{\left\{1\right\}}}^{\left\{2\right\}} \end{array}\right].$$

The final result of network training *y* was the output of the layer {2}: $y^{\left\{2\right\}}$, as in Formula (13) [89]:

(13) $$y=y^{\left\{2\right\}}=f^{\left\{2\right\}}{}_{\mathrm{activ}}\left(W^{\left\{2\right\}}\cdot \left(f^{\left\{1\right\}}{}_{\mathrm{activ}}\left(W^{\left\{1\right\}}\cdot X+B^{\left\{1\right\}}\right)\right)+b_{1}^{\left\{2\right\}}\right).$$

In the last stage, the outputs underwent denormalization so as to express approximated stress.

### 3.3. Choice of Learning Parameters

The selection of learning parameters, such as activation functions, training algorithm, performance function and its goal, learning rate, momentum and others should be in correspondence with the specific data assigned to the learning process and the phenomenon that they represent [90]. Below are the presented choices determined for this study and their justification. The numerical values of the assumed learning parameters are summarized in Table 2.

As for the activation functions, a hyperbolic tangent sigmoid function (Formula (4)) was implemented in the hidden layer {1}. According to [87], tansig is a recommendation for addressing nonlinear problems, and the closed-cell aluminium compression is a nonlinear phenomenon. Additionally, the hyperbolic tangent sigmoid function was successfully verified in preliminary calculations and previous studies on the modelling of open-cell aluminium [49,50]. The activation function for the output layer {2} was the linear activation function—purelin (Formula (10)).

Regarding the training algorithm, the Levenberg–Marquardt procedure was selected [91]. For this procedure the mean square error, as defined in Formula (14), was chosen as the performance function:

(14) $$MSE=\frac{\sum_{i=1}^{n}{\left(t_{i}-o_{i}\right)}^{2}}{n}=\frac{\sum_{i=1}^{n}{\left(e_{i}\right)}^{2}}{n},$$

where:

$t_{i}$—i-th target for the network;
$o_{i}$—i-th output for the network;
i—individual data index;
n—number of all data.

The error was defined as in Expression (15):

(15) $$e_{i}=t_{i}-o_{i}.$$

The performance function’s goal was set as 0, and the minimum performance gradient was assumed as 10^−10^. Based on the former application of neural network modelling to the compressive behaviour of cellular metals [49,50] the number of epochs to train was set as 100,000.

**Figure 5:**
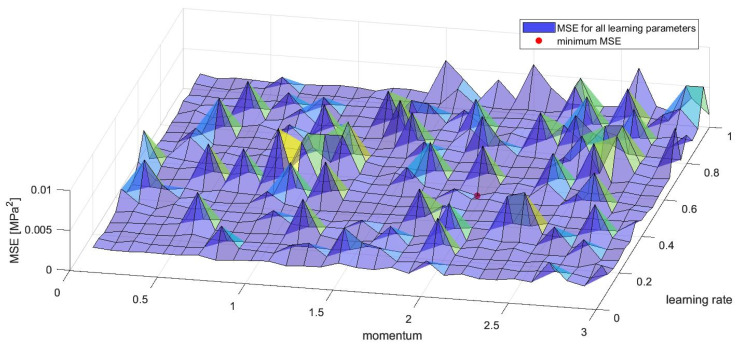
Values of the performance function (MSE) with reference to momentum and learning rate obtained in calibration of these parameters for networks with 12 neurons in the hidden layer.

**Figure A3:**
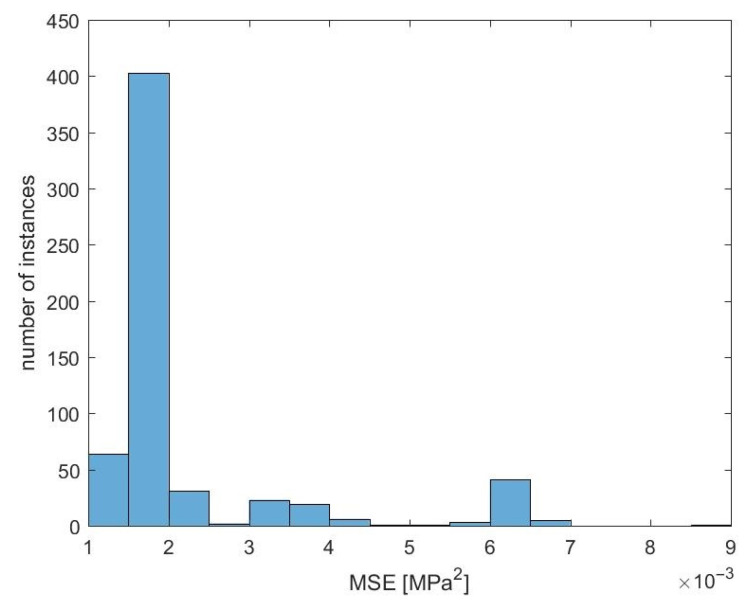
Histogram of MSE in calibration of learning parameters.

The learning rate and momentum were assumed as the result of a specially designed procedure. The procedure consisted of the examination of a number of networks in terms of assigning them various values of these two learning parameters and comparing the obtained values of the performance function (MSE) in each case. Based on the robustness analysis for a related phenomenon of compression in open-cell aluminium [49,50], it was decided that the architecture of examined networks would have the complexity of 12 neurons in the hidden layer. Learning rate values were taken from the range 〈0.05;1.00〉 with the step 0.05. Momentum values were taken from the range 〈0.1;3.0〉 with the step 0.1. Results are shown in Figure 5; the chosen values were 2.0 for momentum and 0.05 for the learning rate, and they occurred for the minimum $MSE_{min}=0.023\;{\mathrm{MPa}}^{2}$. Additional remarks about the presented calibration can be found in Appendix C, Figure A3.

### 3.4. Algorithm for Building and Training Networks

**Figure 6:**
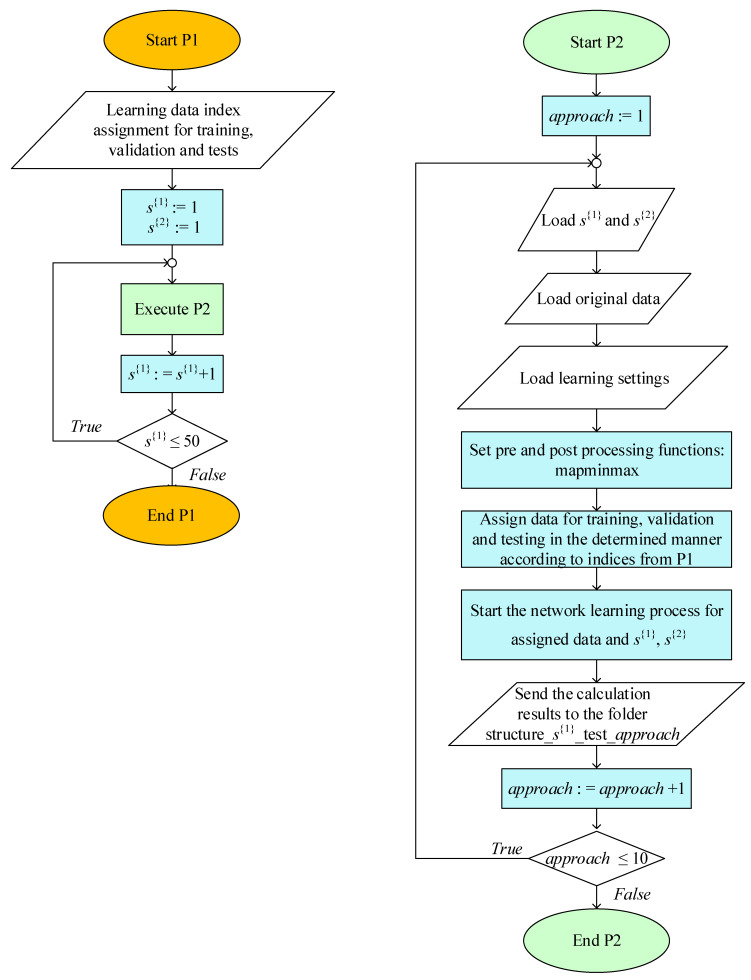
The algorithm for identification of the best possible value of $s^{\left\{1\right\}}$ in the feedforward neural network with one hidden layer model. On the left side: the parent procedure P1; and on the right side: the nested procedure P2.

In order to generate a considerable number of comparable networks that modelled the aluminium foam compression, the algorithm shown in Figure 6 was implemented. The algorithm consisted of two procedures: P1 (parent) and P2 (nested). The main aim of P1 was to provide varying unit network architecture parameters by attributing a given number of neurons $s^{\left\{1\right\}}$ to hidden layers of NNs. The aim of P2 was to build, train, validate and test the given network, which was structured according to parameters from P1, and also to compute measures used in further criterial network evaluation. Please note, that in accordance with the general research concept and the main assumption explained in the beginning of Section 3, the data used in P2 were the subset for 11 out of 12 specimens. In conclusion, the algorithm (P1 + P2) served to build, train and test individual models; however, the first-step collective evaluation was performed later (Section 3.5).

The range of $s^{\left\{1\right\}}$ in P1 was assumed as $s^{\left\{1\right\}}=\langle 1;50\rangle$. Such a range was selected due to the specificity of the data for NN. Additionally, previous studies regarding open-cell metals [49,50] have shown that such an interval allows for additional conclusions about robustness and overfitting [82,87].

In the first iteration of a network learning process the initial values of weights and biases in the first layer are assigned randomly. This means that networks with the same architecture specifications almost certainly lead to different solutions. Taking this fact into account, in the designed algorithm there were not only networks varying in the hidden layer neuron size built but also for each given $s^{\left\{1\right\}}$, and 10 networks were created, trained, validated and tested (procedure P2). These calculations were labelled as approaches and numbered consecutively from 1 to 10. Such repetitions increased the probability of obtaining the minimum of the global performance function [84,89]. An additional advantage of multiple approaches is that they enable the discussion of robustness.

### 3.5. Evaluation Criteria

At this point it should be noted that the choice of learning parameters (mostly the selection of the performance function and its goal, Section 3.3) already imposed certain aspects of the evaluation approach. This is inevitable and ‘internally’ connected to building networks and assuming their structure and learning mode.

As for the evaluation of network results ‘from the outside’, there may be multiple approaches assumed. Two most obvious paths are: one may expect the network to either provide the most accurate outputs or to provide results in a short time, which also applies to a simpler model. Additionally, the repeatability of results may play a role. In general, then, balancing between—or combining—different evaluation strategies is what designers choose most often, and so the same was implemented in the present research. A formal description of the mentioned evaluation approaches and mathematical expressions for the respective criteria are given in Section 3.5.1, Section 3.5.2, Section 3.5.3 and Section 3.5.4.

In the present research, the complexity of the structure of the neural network consists of the number of neurons in the hidden layer {1}. The parameter which characterizes this complexity is $s^{\left\{1\right\}}$. As was scrupulously explained in Section 3.2, this parameter decides the sizes of the matrix $W^{\left\{1\right\}}$ and the vectors $B^{\left\{1\right\}}$ and $W^{\left\{2\right\}}$. So, one can say that the number of neurons in the hidden layer characterizes the modelled phenomenon together with the data assigned to the learning process. The aim of the evaluation in the present study, then, is to choose such an $s^{\left\{1\right\}},$ which would provide the most appropriate model.

#### 3.5.1. The Idea of a Two-Step Evaluation

This study was designed to conduct a two-step evaluation (compare: the key assumption described in the beginning of Section 3), which will now be explained thoroughly.

The networks built and trained in the algorithmic computations were particular models of the phenomenon of the compression of 11 physical objects composed of closed-cell aluminium. Part of the experimental data for these specimens was devoted to learning: 3/5 of data to the training stage and 1/5 of data to the validation stage. The remaining 1/5 of data were devoted to testing the model against unknown information, which still concerned the 11 specimens. Results from the test stage were the subject of the first-step evaluation. This evaluation allowed for a collective view of all particular models and the choice of the most appropriate model of the compression of the 11 specimens.

The following step consisted of exposing the trained networks obtained from the algorithm to data for another physical object—the 12th sample. Results of this mapping were subject to the second-step evaluation. This time the performed evaluation allowed for the assessment of whether the networks could be used not only as particular models but also as general models capable of prognosis. If the answer was positive, the second-step evaluation also allowed for choosing the best general model.

Such a design of the evaluation stages reflects the bias of the data that we intentionally wanted to introduce; by the assumption of the relation type expressed in Formula (2) it was assumed that apparent density affected the response of aluminium foam subjected to compression. This was the basis for holding one specimen away, so that the prediction potential of the given model was verified with respect to the new apparent density value formed outside the values that the model would ‘know’.

#### 3.5.2. Accuracy of Outputs, Overfitting

Accuracy and overfitting are two sides of the same coin, and the criteria to assay them may be formulated analogously. In the used approach, accuracy would be assessed in the first-step evaluation and overfitting in the second-step evaluation.

In the first case the mean absolute relative error calculated for the network testing stage obtained for the given approach was chosen as the measure used for the formulation of the assessment criterion [92]. The criterion reads: the minimal value of all mean absolute relative errors obtained for all architectures and all approaches from the test stage is the indicator of the ‘best’ network. In other words, it indicates the particular model with $s^{\left\{1\right\}}{}_{\mathrm{best}}$ neurons in the hidden layer and trained at the particular $approach_{\mathrm{best}}$, for which the condition is fulfilled. Such a formulated main criterion is symbolically expressed as in Formula (16):

(16) $$Crit1_{1}=\min\left\{{\left(MARE_{\mathrm{Test}}\right)}_{s^{\left\{1\right\}},approach}\right\},$$

where:

$Crit1_{1}$—value of the measure assumed for Criterion 1 used for the first-step evaluation;
$s^{\left\{1\right\}}$—given number of neurons in the hidden layer;
approach—given number of repetitions of the network learning for the given network architecture;
${\left(MARE_{\mathrm{Test}}\right)}_{s^{\left\{1\right\}},approach}$—maximum absolute relative error obtained for the testing stage, according to the Formula (17):

(17) $${\left(MARE_{\mathrm{Test}}\right)}_{s^{\left\{1\right\}},approach}=\mathrm{mean}\left\{{\left(\left|\frac{t_{i\mathrm{Test}}-o_{i\mathrm{Tes}t}}{t_{i\mathrm{Test}}}\right|\right)}_{s^{\left\{1\right\}},approach}\right\},$$

where:

$t_{i\mathrm{Tes}t}$—i-th target for the network in the testing stage;
$o_{i\mathrm{Test}}$—i-th output for the network in the testing stage;
i—individual data index, should exhaust all data.

The above criteria for the best accuracy should be complemented by an additional criterion to prevent the choice of the model, which is overfitted. That is, to prevent the situation in which the chosen best network memorized the data instead of working out relations hidden in the data. Such a network would not be capable of prognosis, so it could not be used as a general model. For this reason, the second-step evaluation was proposed, in which results of the verification of the network were analyzed against data previously unknown to it. Again, a mean absolute relative error was chosen as the criterion measure. The criterion takes the form symbolically written in Formula (18):

(18) $$Crit1_{2}={\left(MARE_{\mathrm{Verif}}\right)}_{s^{\left\{1\right\}}{}_{\mathrm{best}},approach_{\mathrm{best}}}\leq Crit1_{2.\mathrm{threshold}},$$

where:

$Crit1_{2}$—value of the measure assumed for Criterion 1 used for the second-step evaluation;
${\left(MARE_{\mathrm{Verif}}\right)}_{s^{\left\{1\right\}}{}_{\mathrm{best}},approach_{\mathrm{best}}}$—mean absolute relative error from the verification of the network with the given $s^{\left\{1\right\}}{}_{\mathrm{best}}$ and taught in the given $approach_{\mathrm{best}}$ against external data;
$Crit1_{2.\mathrm{threshold}}$—threshold for Criterion 1 used for the second-step evaluation;

In cases where accuracy is particularly important one may demand that:

(19) $$Crit1_{2.\mathrm{threshold}}\approx Crit1_{1}.$$

In the event of considerable overfitting, it would not be possible to fulfil Expression (19). One would then iteratively verify networks respective to next consecutive local minima among the set $\left\{{\left(MARE_{\mathrm{Test}}\right)}_{s^{\left\{1\right\}},approach}\right\}$ until Condition (19) is met.

There might be more detailed demands imposed on the outputs, e.g., that outputs are equally credible in the whole mapping range or that none of the absolute relative errors exceed a certain value. In such cases one might choose other or additional measures as auxiliaries in evaluation criteria. Such a measure could be, for example, the maximum absolute relative error obtained for the network with the given number of neurons in the hidden layer and in the given approach, ${\left(MaxARE\right)}_{s^{\left\{1\right\}},approach}$, as in Formula (20):

(20) $${\left(MaxARE\right)}_{s^{\left\{1\right\}},approach}=\max\left\{{\left(\left|\frac{t_{i}-o_{i}}{t_{i}}\right|\right)}_{s^{\left\{1\right\}},approach}\right\},$$

where all symbols at the right side of the equation are defined as in Relations (14,17).

#### 3.5.3. Speed of Calculations

When balancing model complexity and computation time is needed, one should note that there are three main circumstances that affect the speed of network operation: computation capacity of the computing machine, precision of significant numbers (also depending on the data format) and complexity of calculations resulting from the size of the network structure. So, from the viewpoint of the network design, the crucial factor is the minimum network complexity that assures satisfactory outputs. The criterion for finding the minimum sufficient number of neurons $s^{\left\{1\right\}}{}_{\min}$ (and the assigned $approach_{\min}$) might be to demand that the assumed measure, let it be mean absolute relative error from the test stage, does not exceed a certain level. Mathematically, in the first-step evaluation, the criterion reads as in Expression (21) below:

(21) $$Crit2_{1}={\left(MARE_{\mathrm{Test}}\right)}_{s^{\left\{1\right\}},approach}\leq Crit2_{1.\mathrm{threshold}},$$

where:

$Crit2_{2}$—value of the measure assumed for Criterion 2 used for the first-step evaluation;
$Crit2_{2.\mathrm{threshold}}$—threshold for Criterion 2 used for the first-step evaluation;
$s^{\left\{1\right\}}$, approach and ${\left(MARE_{\mathrm{Test}}\right)}_{s^{\left\{1\right\}},approach}$—defined as in Formulas (16) and (17).

For the second-step evaluation this could be formulated in Condition (22) below:

(22) $$Crit2_{2}={\left(MARE_{\mathrm{Verif}}\right)}_{s^{\left\{1\right\}}{}_{\min},approach_{\min}}\leq Crit2_{2.\mathrm{threshold}},$$

where:

Crit2—value of the measure assumed for Criterion 2 used for the second-step evaluation;
${\left(MARE_{\mathrm{Verif}}\right)}_{s^{\left\{1\right\}}{}_{\mathrm{best}},approach_{\mathrm{best}}}$—mean absolute relative error from the verification of the network with the given $s^{\left\{1\right\}}{}_{\min}$ and taught in the given $approach_{\min}$ against external data;
$Crit2_{2.\mathrm{threshold}}$—threshold for Criterion 2 used for the second-step evaluation.

In the case Condition (22) is not satisfied, one should then verify networks with an increased number of neurons in the hidden layer or agree to lower the accuracy demand by increasing the threshold until Expression (22) is met.

The assumption of the mean absolute relative error value as the criterion measure is not the only possibility. In the case the designer or user is more interested in obtaining results which have the same reliability in the whole interval or having no errors (not only the mean) that exceed the assumed threshold, they could select another measure, such as the maximum absolute relative error, as in Definition (20).

#### 3.5.4. Robustness

Balancing accuracy and computation speed does not exploit the network evaluation problem. Obtained networks should also be verified with regard to robustness, which can be understood as insensitiveness to the randomness of the initial bias and weight assumptions. One would be searching for such a number of neurons in the hidden layer, for which, regardless of the random parameters, repeatability of the results is ensured [93]. Analysis of robustness may indicate more than one specific network complexity $s^{\left\{1\right\}}{}_{\mathrm{robust}}=\left\{s^{\left\{1\right\}}{}_{\mathrm{robust},j}\right\}$, which would comply with the demand of repeatability.

In the present research the sum of absolute errors from validation is assumed as the measure for robustness criterion [94]. This criterion is expressed in Formula (23) below:

(23) $$Crit3=\left|{\left(SAE_{\mathrm{Valid}}\right)}_{s^{\left\{1\right\}},approach}-\frac{1}{k}\sum_{approach=1}^{k}{\left(SAE_{\mathrm{Valid}}\right)}_{s^{\left\{1\right\}},approach}\right|\leq Crit3_{\mathrm{threshold}},\;\mathrm{for}\;\mathrm{all}\;approach\in \langle 1;k\rangle ,$$

where:

Crit3—value of the measure assumed for Criterion 3;
k—total number of approaches for the given $s^{\left\{1\right\}}$;
$Crit3_{\mathrm{threshold}}$—threshold for Criterion 3;
${\left(SAE_{\mathrm{Valid}}\right)}_{s^{\left\{1\right\}},approach}$—as in Formula (24):
  (24) $$SAE=\sum_{i=1}^{n}\left(t_{i}-o_{i}\right)=\sum_{i=1}^{n}e_{i},$$
  where remaining symbols are denoted as in (14,15).

The strict demand would be to assume that the threshold is near or equal to zero:

(25) $$Crit3_{\mathrm{threshold}}\approx 0.$$

However, a strongly posed demand such as Demand (25) is not necessary in all cases. Moreover, there would be instances for which a lighter condition would be justified: only limited (23) and not necessarily (25). This would include cases for which for ‘only’ a considerable majority of approaches for the given $s^{\left\{1\right\}}{}_{\mathrm{robust},j}$ would meet (23) and a negligible number of networks would not. In some cases, this would also be acceptable solution. Let it be expressed it as Alternative Criterion 3 (26) below:

(26) $$\begin{array}{c} AltCrit3=\left|{\left(SAE_{\mathrm{Valid}}\right)}_{s^{\left\{1\right\}},approach}-\frac{1}{k}\sum_{approach=1}^{k}{\left(SAE_{\mathrm{Valid}}\right)}_{s^{\left\{1\right\}},approach}\right|\leq AltCrit3_{\mathrm{threshold}},\\ \mathrm{for}\;M\;\mathrm{of}\;\mathrm{all}\;approach\in \langle 1;k\rangle , \end{array}$$

where:

AltCrit3—value of the measure assumed for Alternative Criterion 3;
$AltCrit3_{\mathrm{threshold}}$—threshold for Alternative Criterion 3, which may also not necessarily be assumed as 0;
M—number or percentage value for total approaches that must comply with Condition (26);
other symbols—as defined in (23).

## 4. Results and Discussion

As was said in Section 3.1.2 and Section 3.5.1, experimental data from the compression of 12 specimens were divided into two sets: the data of 11 specimens were devoted to building particular models, and their first-step evaluation and the data of one sample were left aside for external verification in the second-step evaluation. Sample Z_14_p was selected for the external verification. In consequence, the results are now presented as follows:

- Section 4.1 will give results from the validation stage from the training of networks (11 sample data set).
- Section 4.2 will be devoted to choosing the most adequate network according to criteria of the first- and second-step evaluation and thus will show results from the test stage of teaching networks (11 sample data set) as well as from the verification of networks against external data (specimen Z_14_p).
- Section 4.3 will present detailed results for the final chosen networks.

Please note that the colour and notification convention is common for all figures presented in Section 4 and Appendix D. The convention will be explained in detail by the description of mean square error results in Section 4.1 and later on is applied analogously and treated as known.

### 4.1. Internal Network Evaluation and Robustness

**Figure 7:**
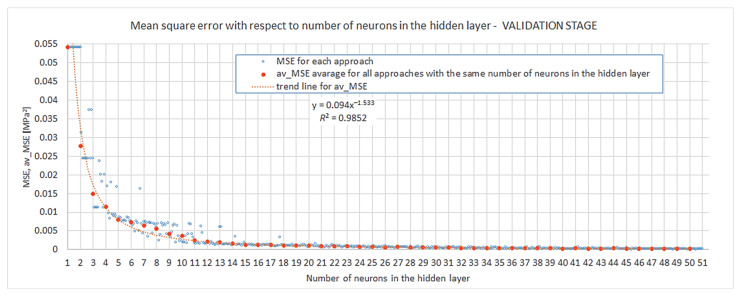
Mean square error from the validation stage.

It was assumed that the performance function for the analyzed networks was the mean square error MSE, Definition (14). The goal for this function was set as 0. In Figure 7 below, there are the presented results obtained for the performance function at the validation stage for all networks. Individual mean square errors are depicted as hollow blue dots. Additionally, there are solid orange dots in the plot denoted as av_MSE, which refer to the arithmetic average of MSEs obtained for all approaches for the given number of neurons in the hidden layer $s^{\left\{1\right\}}$. A trend line for magnitude av_MSE is also shown (dashed orange line).

The general conclusion drawn from these results might be that modelling of the compression of closed-cell aluminium with networks of increasing complexity of the hidden layer is not a chaotic but an ordered phenomenon. The relation between complexity and convergence, understood as nearing to the achievement of the performance function’s goal, can be very well described by a power law (determinacy coefficient for such a relation was obtained as $R^{2}=0.9852$).

**Figure 8:**
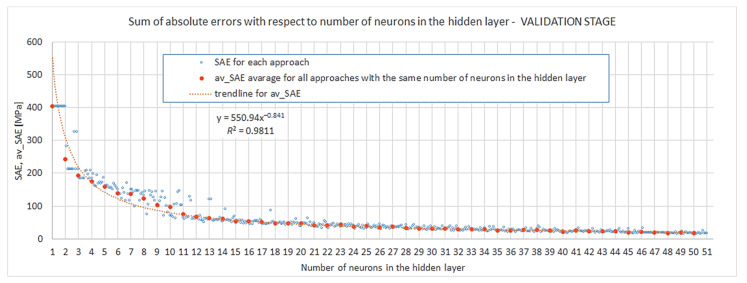
Sum of square errors from the validation stage.

The problem of robustness will now be discussed. Looking at Figure 8 and taking into account Criterion 3 (23) and Demand (25), one can observe that the difference between $SAE_{\mathrm{Valid}}s$ from individual es and the average of all approaches for the given $s^{\left\{1\right\}}$ tends to be zero for all instances of the approach for $s^{\left\{1\right\}}{}_{\mathrm{robust}}\geq 18$. We can also slightly alleviate the robustness condition and utilize Alternative Criterion 3 (26) with a threshold near 0, assuming $M_{9}=9$ of 10 approaches or $M_{8}=8$ of 10 approaches. In these cases, we read from Figure 8 that $s^{\left\{1\right\}}{}_{\mathrm{robust}.M9}\geq 14$ and $s^{\left\{1\right\}}{}_{\mathrm{robust}.M8}\geq 11$.

The results are in agreement with the pre-assumption determined at the stage of the choice of learning parameters. At that phase the influence of learning rate and momentum on the training process of networks with $s^{\left\{1\right\}}=12$ neurons in the hidden layer was evaluated. Such a choice fits Alternative Criterion 3 (26) with the parameter $M_{8}=8$.

### 4.2. Choice of the Most Appropriate Network Specifications

**Figure 9:**
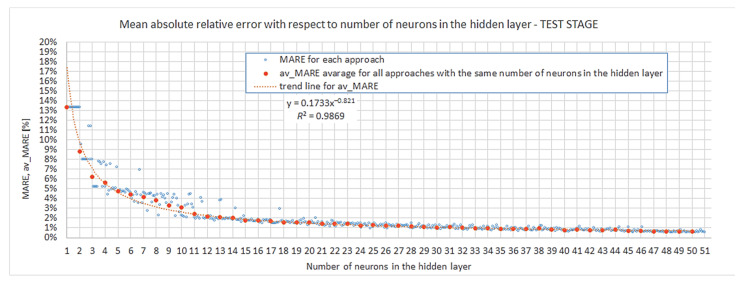
Mean absolute relative error from the test stage.

**Figure 10:**
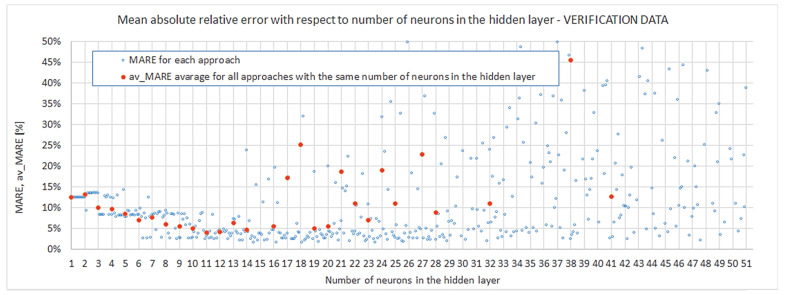
Mean absolute relative error from verification of external data.

**Figure A4:**
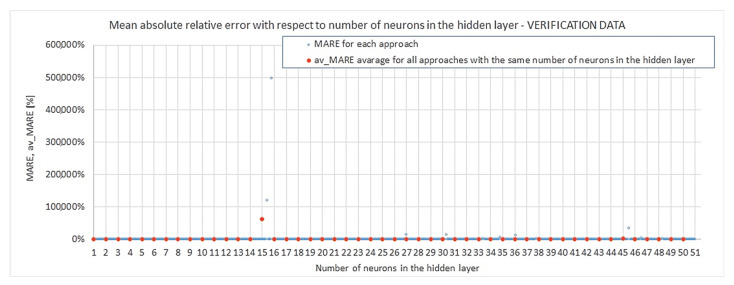
Mean absolute relative error from verification of external data.

In general, we are looking for the most appropriate number of neurons in the hidden layer that would guarantee the desired outputs’ quality with regard to the assumed criteria. The total interval of $s^{\left\{1\right\}}$ in the studied algorithm was $s^{\left\{1\right\}}=\langle 1;50\rangle$. In Section 4.1 it was already stated that this interval should be narrowed to an $s^{\left\{1\right\}}$ less than 18 or 14 or 11 neurons, depending on the level of the desired results’ repeatability. These boundaries should be taken into account in the evaluation of models. Both the first- and second-step evaluation are referred to in Figure 9 and Figure 10 below. Figure 9 shows the mean absolute relative error from the test stage of training networks, $MARE_{\mathrm{Test}}$, with respect to the size of the hidden layer. Figure 10 gives the mean absolute relative error from the verification of trained networks (particular models) against external data, $MARE_{\mathrm{Verif}}$, with respect to the size of the hidden layer. Please note that the vertical axis in Figure 10 was scaled. Due to this graphic processing, some of the results could not fit in the plots. This was performed in order to present the results clearly and legibly in the range of the hidden layer neuron number important for the discussion. The omission of some of the results in these plots did not affect reasoning or conclusions. In Appendix D we include the respective graph (Figure A4), which gives all obtained results without vertical axis scaling.

#### 4.2.1. Most Accurate Outputs, Overfitting

In the first-step evaluation, according to Criterion (16) for accuracy, the minimum value of $MARE_{\mathrm{Test}}$ and the respective network structure for it (identified by $s^{\left\{1\right\}}{}_{\mathrm{best}},\;approach_{\mathrm{best}}$) are sought. Table 3 presents results found in this search. The complexity of the ‘best’ network was 48 neurons in the hidden layer, which is far beyond the boundaries set in the analysis of robustness. The application of the second-step evaluation shows that for this network overfitting was on the unsatisfactory level (the last column of Table 3), and though the particular model was the best in terms of accuracy, it cannot be used for prognosis. One could iteratively search for consequent minima in $Crit1_{1}$, but judging from the lack of diversity in the results until the limit of robustness, this path would be too inefficient to follow. Instead, we proceed to the alternative approach described in Section 3.5.2.

#### 4.2.2. Outputs in Terms of Increasing Speed of Calculations

In this analysis Criterion (21) and (22) were used with the mean absolute relative errors chosen as the measures $MARE_{\mathrm{Test}},MARE_{\mathrm{Verif}}$, respectively, in the first- and second-step evaluation. It was decided that several threshold values in Criterion (21) would be assumed in the first-step evaluation, so multiple indications of $s^{\left\{1\right\}}{}_{\min}$ and the respective $\;approach_{\min}$ were obtained. Then Criterion (22) was applied in the second-step evaluation to the indicated models. Results from this evaluation are summarized in Table 4.

Looking at Figure 9, one notices that all particular models with $s^{\left\{1\right\}}\geq 5$ (except one) produced a mean absolute relative error on a good engineering accuracy level of less than $MARE_{\mathrm{Test}}\leq 5\%$. The 5% threshold had already been obtained for the first time by a particular model with four neurons in the hidden layer. The simplest network $\left[s^{\left\{1\right\}}{}_{\min},\;approach_{\min}\right]=\left[4,2\right]$ provides a relatively good particular model but gives an almost two-times-greater mean absolute relative error when it comes to prognosis. Distinctively good results were obtained for the network $\left[s^{\left\{1\right\}}{}_{\min},\;approach_{\min}\right]=\left[6,6\right]$; errors obtained in the verification of prognosing the capability of the model were even better than in the particular model itself. It should be noted that all the remaining networks listed in Table 4 exhibited $MARE_{\mathrm{Verif}}\leq 5\%$, which is a good engineering accuracy level for prognosis capability. Lastly, none of the analyzed particular models achieved a better accuracy in prognosis than $MARE_{\mathrm{Verif}.\min}=1.661\%$ (the network $\left[s^{\left\{1\right\}}{}_{\min},\;approach_{\min}\right]=\left[14,5\right]$). This could mean that it is extremely difficult to obtain a model capable of a more accurate prognosis without changing the structure or learning parameter assumptions and that at least such a value of error is inevitable. One more observation should be noted: Figure 10 shows that for networks with ≥13 neurons, instances of approaches with considerable overfitting already start to occur.

The networks with four and six neurons in the hidden layer, distinguished in the previous paragraph, do not fall into the intervals, which assures robustness ($s^{\left\{1\right\}}{}_{\mathrm{robust}}\geq 18$, $s^{\left\{1\right\}}{}_{\mathrm{robust}.M9}\geq 14$, $s^{\left\{1\right\}}{}_{\mathrm{robust}.M8}\geq 11$). This condition is fulfilled by the model $\left[s^{\left\{1\right\}}{}_{\min},\;approach_{\min}\right]=\left[11,4\right]$, for which $MARE_{\mathrm{Test}}\leq 2\%$ and $MARE_{\mathrm{Verif}}\leq 3\%$. These two values confirm that the particular model here is very good in terms of accuracy, and it also has the ability to provide high-quality prognoses. The probability of obtaining similar a quality in a model in repeating network training is sufficient ($s^{\left\{1\right\}}{}_{\mathrm{robust}.M8}\geq 11$).

In Section 4.2. the analysis of robustness was presented. Quite strict demands, including $Crit3_{\mathrm{threshold}}\approx 0$ and $AltCrit3_{\mathrm{threshold}}\approx 0$ in Conditions (23) and (26), respectively, were assumed. Nevertheless, one does not have to be that rigorous. Let us now complement the analysis of repeatability, but for the stage of the first- and second-step evaluation. This requires the introduction of another measure: av_MARE—the arithmetic average of the MAREs obtained for all approaches for the given number of neurons in the hidden layer $s^{\left\{1\right\}}$:

(27) $$\mathrm{av}\_MARE=\frac{1}{k}\sum_{approach=1}^{k}\left(MARE_{s^{\left\{1\right\}},approach}\right),$$

where:

$s^{\left\{1\right\}}$—given fixed number of neurons in the hidden layer;
k—total number of approaches for the given $s^{\left\{1\right\}}$; here k=10.

In the first-step evaluation the measure defined in Formula (27) is the average mean absolute relative error for the test stage in training of the given network ($\mathrm{av}\_MARE_{\mathrm{Test}}),$ and in the second-step evaluation it is the mean absolute relative error from the verification of the given network against external data $\left(\mathrm{av}\_MARE_{\mathrm{Verif}}\right)$.

By application of Measure (27) in Criterion (21), it was possible to indicate the number of neurons in the hidden layer for which the new measure complied with the assumed thresholds $s^{\left\{1\right\}}{}_{\min.\mathrm{av}.M.T.}$ Results are summarized in the first three columns of Table 5. The results show that if we regard the average results of all approaches for a given $s^{\left\{1\right\}}$, $\mathrm{av}\_MARE_{\mathrm{Test}}\leq 5\%$ is fulfilled already for five neurons in the hidden layer. We also found confirmation that networks with at least 11 neurons in the hidden layer have very good accuracy on average: $\mathrm{av}\_MARE_{\mathrm{Test}}\leq 2.5\%$.

The average mean absolute error was also substituted in Criterion (22) in the second-step evaluation. This time, however, the criterion thresholds had to be elevated. They are listed in Table 5 together with corresponding minimum numbers of neurons in the hidden layer $s^{\left\{1\right\}}{}_{\min.\mathrm{av}.M.V.}$ and obtained values of the criterion measure in columns 4–6. The first thing that catches attention is that none of the networks achieved $\mathrm{av}\_MARE_{\mathrm{Test}}\leq 4\%$, and only the result for $s^{\left\{1\right\}}{}_{\min.\mathrm{av}.M.V.}=11$ is slightly above this limit. It turns out that the criterion measure value obtained for networks with 11 neurons in the hidden layer was the global minimum for $s^{\left\{1\right\}}{}_{\min.\mathrm{av}.M.V.}\in \langle 1,50\rangle$. This once again shows that such model complexity produces good-quality outputs with relatively low errors both in the particular model as well as in prognosis. Lower complexity networks do not satisfy the engineering precision threshold of 5%.

### 4.3. Results for Optimal Networks

Considerations presented in Section 4.1 and Section 4.2 allowed for choosing particular networks to finally show as examples of how the structure and learning parameters of neural network models influence the quality of the description of the phenomenon of the compression of aluminium foams. We selected the following networks:

1. The network $\left[s^{\left\{1\right\}}{}_{\min},\;approach_{\min}\right]=\left[4,2\right]$ is the least complex structure, but still provides acceptable accuracy itself ${\left(MARE_{\mathrm{Test}}\right)}_{4,2}=4.455\%$ and for prognosis ${\left(MARE_{\mathrm{Verif}}\right)}_{4,2}=8.688\%$; however, four neurons do not guarantee robustness.
2. The network $\left[s^{\left\{1\right\}}{}_{\min},\;approach_{\min}\right]=\left[6,6\right]$ is still a relatively simple structure but assures good accuracy itself ${\left(MARE_{\mathrm{Test}}\right)}_{6,6}=3.572\%$ and for prognosis ${\left(MARE_{\mathrm{Verif}}\right)}_{6,6}=2.689\%$; but six neurons do not guarantee robustness.
3. The network $\left[s^{\left\{1\right\}}{}_{\min},\;approach_{\min}\right]=\left[11,4\right]$ is a relatively complex structure; however, it shows very good accuracy on many levels, including ${\left(MARE_{\mathrm{Test}}\right)}_{11,4}=1.959\%$ and for prognosis ${\left(MARE_{\mathrm{Verif}}\right)}_{11,4}=2.976\%$; also, 11 neurons are within the boundary of 80% robustness.
4. The network $\left[s^{\left\{1\right\}}{}_{\mathrm{best}},\;approach_{\mathrm{best}}\right]=\left[48,10\right]$ is a very complex structure, showing extremely good particular accuracy ${\left(MARE_{\mathrm{Test}}\right)}_{48,10}=0.507\%$ and very adverse overfitting in prognosis ${\left(MARE_{\mathrm{Verif}}\right)}_{48,10}=35.049\%$; 48 neurons are very safe in terms of robustness.

**Figure 11:**
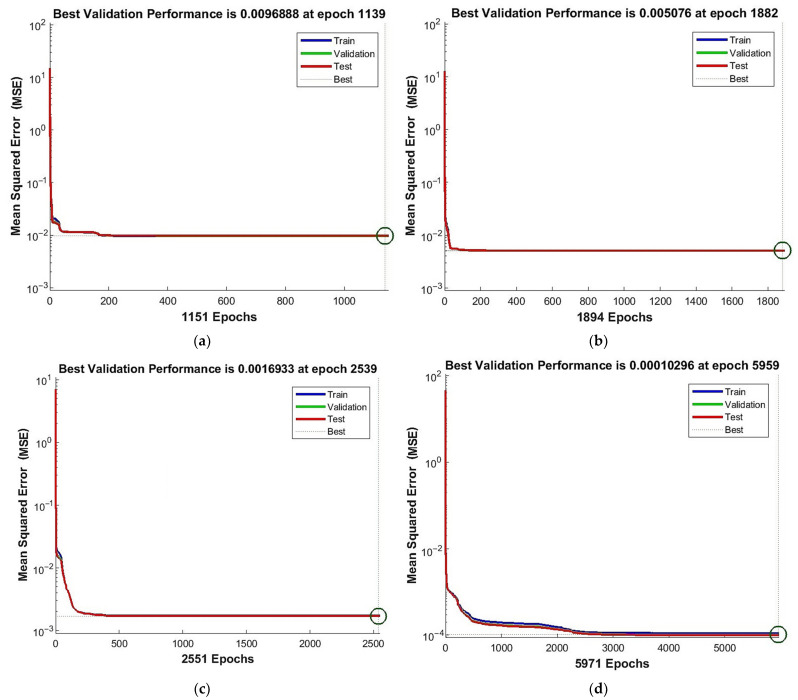
Performance function’s course: (**a**) network $\left[s^{\left\{1\right\}}{}_{\min},\;approach_{\min}\right]=\left[4,2\right]$; (**b**) network $\left[s^{\left\{1\right\}}{}_{\min},\;approach_{\min}\right]=\left[6,6\right]$; (**c**) network $\left[s^{\left\{1\right\}}{}_{\min},\;approach_{\min}\right]=\left[11,4\right]$; and (**d**) network $\left[s^{\left\{1\right\}}{}_{\mathrm{best}},\;approach_{\mathrm{best}}\right]=\left[48,10\right]$.

The performance function’s course for the above networks is presented in Figure 11. Results of the least mean square error MSE together with the epoch, for which they were attained, are located at the top of each plot and in Table 6.

It can be observed that the structure with 11 neurons needs about two times more operations than the structure with four neurons. Additionally, the very complex structure with 48 neurons needs about five times more operations than the simplest network. On the other hand, the minimum of the mean square error is about five times smaller for the network [11,4] than for [4,2] and hundred times smaller for the most complex one with respect to the simplest one. Taking into consideration these results, one notices that 11 neurons in the hidden layer provide satisfactory performance results still at a relatively low cost of calculation time.

**Figure 12:**
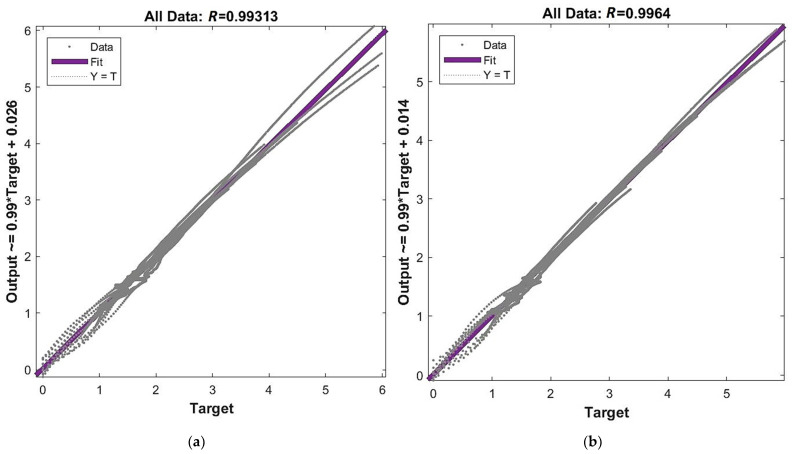

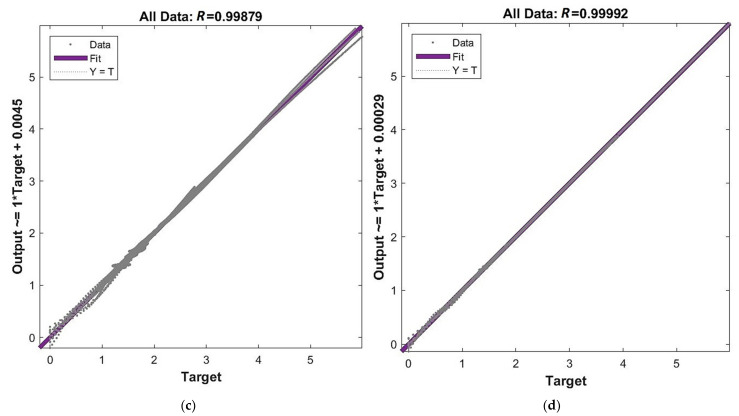
Regression for all training stages: (**a**) network $\left[s^{\left\{1\right\}}{}_{\min},\;approach_{\min}\right]=\left[4,2\right]$; (**b**) network $\left[s^{\left\{1\right\}}{}_{\min},\;approach_{\min}\right]=\left[6,6\right]$; (**c**) network $\left[s^{\left\{1\right\}}{}_{\min},\;approach_{\min}\right]=\left[11,4\right]$; and (**d**) network $\left[s^{\left\{1\right\}}{}_{\mathrm{best}},\;approach_{\mathrm{best}}\right]=\left[48,10\right]$.

**Figure A5:**
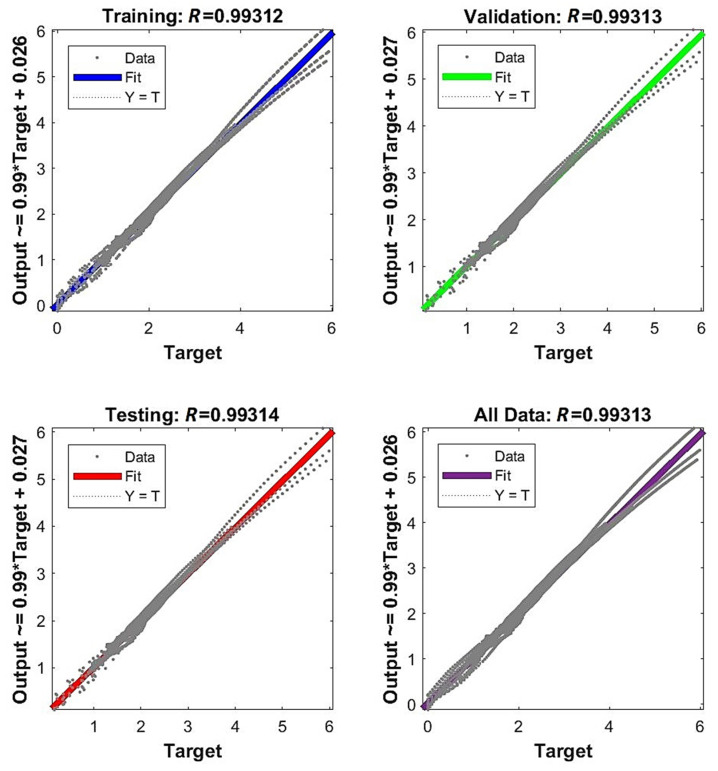
Regression for all stages of network training (training, validation, test) and for the three stages cumulatively for the network $\left[s^{\left\{1\right\}}{}_{\min},\;approach_{\min}\right]=\left[4,2\right]$.

**Figure A6:**
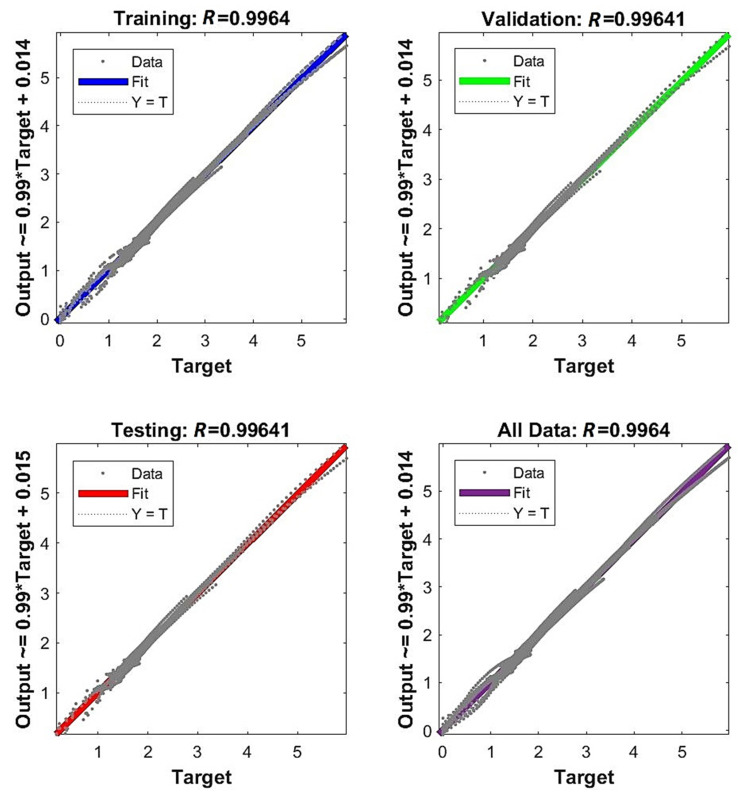
Regression for all stages of network training (training, validation, test) and for the three stages cumulatively for the network $\left[s^{\left\{1\right\}}{}_{\min},\;approach_{\min}\right]=\left[6,6\right]$.

**Figure A7:**
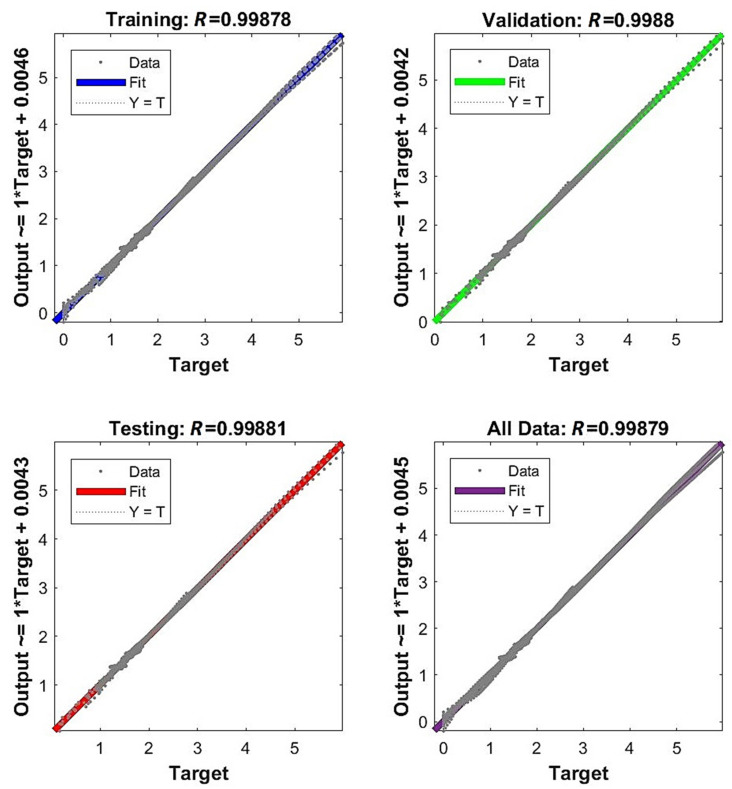
Regression for all stages of network training (training, validation, test) and for the three stages cumulatively for the network $\left[s^{\left\{1\right\}}{}_{\min},\;approach_{\min}\right]=\left[11,4\right]$.

**Figure A8:**
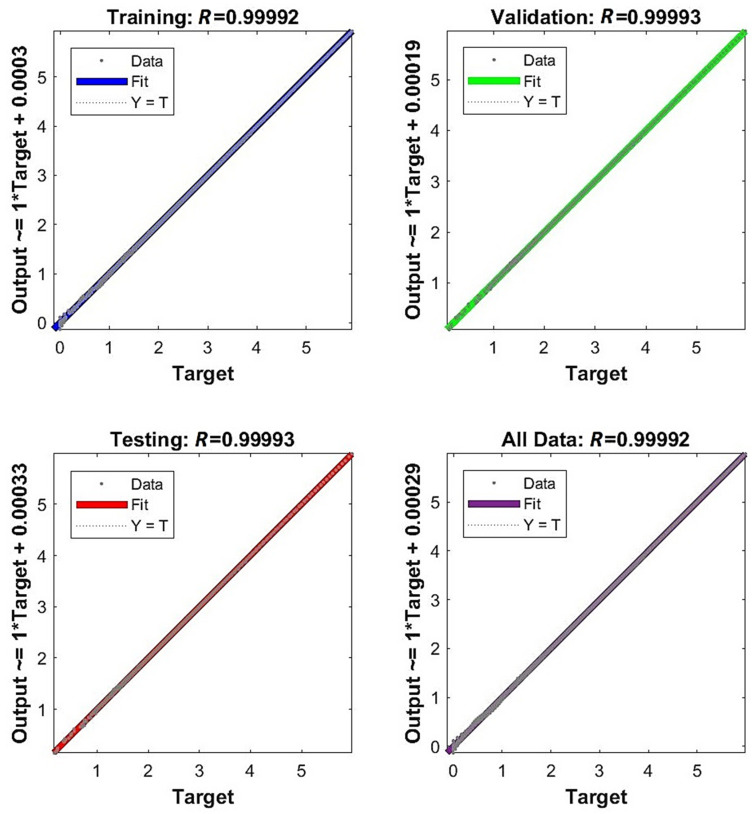
Regression for all stages of network training (training, validation, test) and for the three stages cumulatively for the network $\left[s^{\left\{1\right\}}{}_{\min},\;approach_{\min}\right]=\left[48,10\right]$.

Figure 12 below presents plots of regression for joined stages of network training (training + validation + test). Values of the Pearson coefficient are shown at the top of each graph. Equations for the linear regression line are given at the left side of each graph. These plots are supplemented by the regression of each training stage separately, but those graphs were moved to Appendix E: Figure A5, Figure A6, Figure A7 and Figure A8. One can observe that Pearson’s coefficient increases together with the increase in network complexity. However, all results are R≥0.99, which means that all particular models provide very good correlation between outputs and targets.

**Figure 13:**
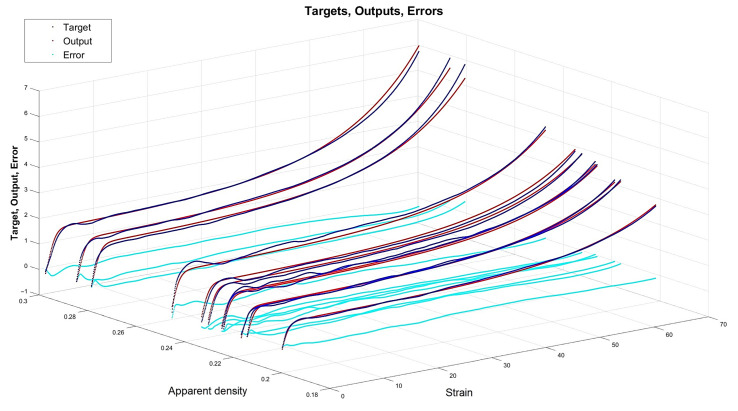
Particular model $\left[s^{\left\{1\right\}}{}_{\min},\;approach_{\min}\right]=\left[4,2\right]$ (red dots). Additionally, errors (light blue) and targets (dark blue) are given.

**Figure 14:**
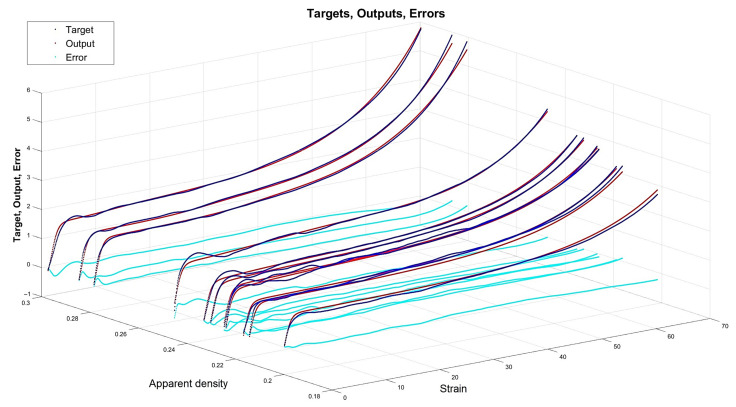
Particular model $\left[s^{\left\{1\right\}}{}_{\min},\;approach_{\min}\right]=\left[6,6\right]$ (red dots). Additionally, errors (light blue) and targets (dark blue) are given.

**Figure 15:**
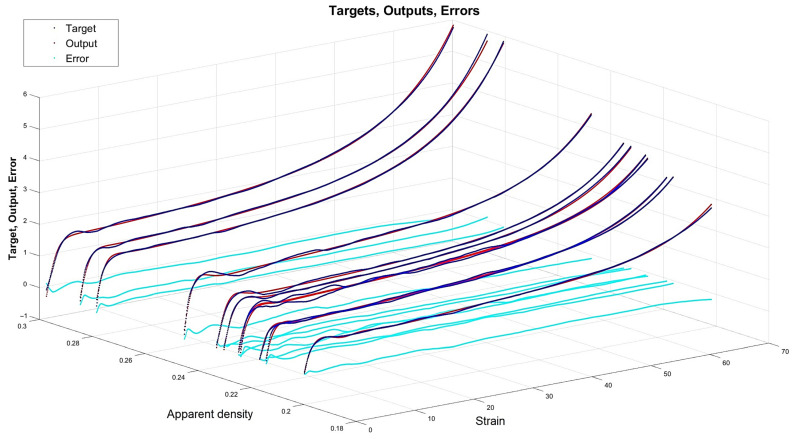
Particular model $\left[s^{\left\{1\right\}}{}_{\min},\;approach_{\min}\right]=\left[11,4\right]$ (red dots). Additionally, errors (light blue) and targets (dark blue) are given.

**Figure 16:**
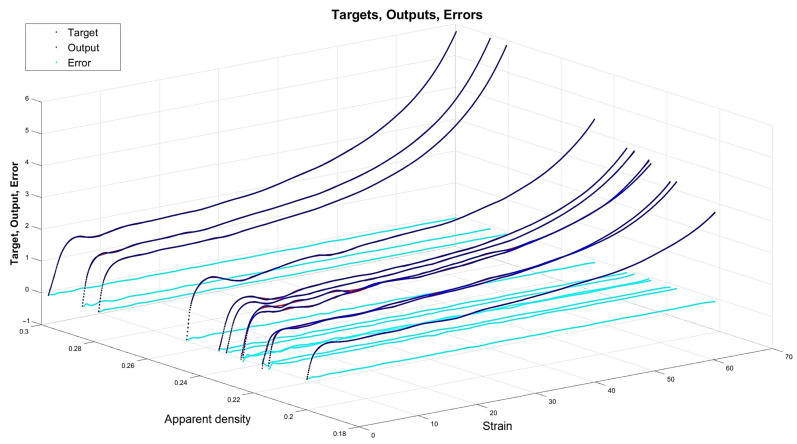
Particular model $\left[s^{\left\{1\right\}}{}_{\min},\;approach_{\min}\right]=\left[48,10\right]$ (red dots). Additionally, errors (light blue) and targets (dark blue) are given.

Figure 13, Figure 14, Figure 15 and Figure 16 depict the four chosen particular models (red dots), errors (light blue) and targets (dark blue). Those plots allow one to see how all individual outputs and targets relate. For the networks with 4, 6 and 11 neurons in the hidden layers we observed satisfactory quality, while the particular network with 48 neurons in the hidden layer maps targets almost perfectly.

**Figure 17:**
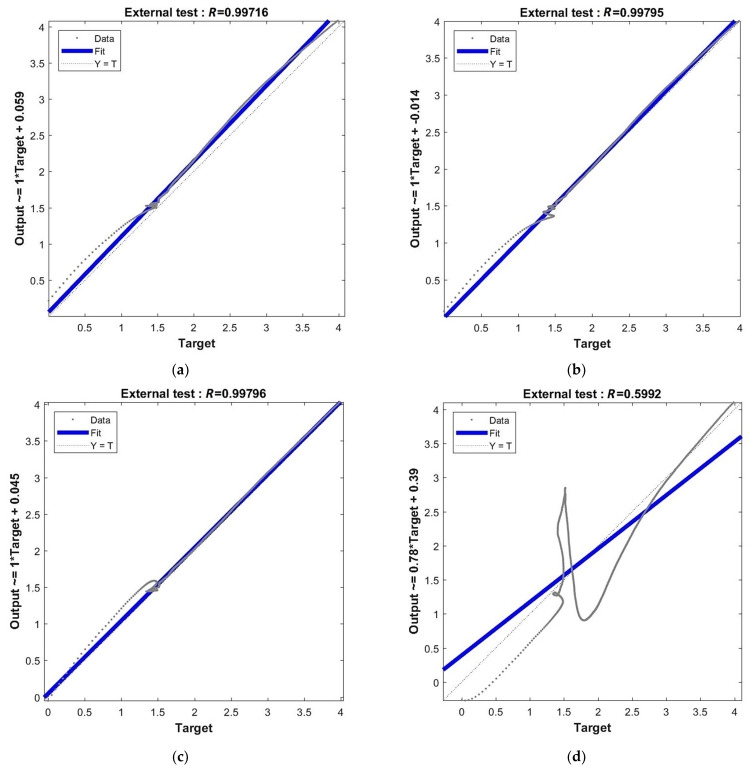
Regression for verification of external specimen data: (**a**) network $\left[s^{\left\{1\right\}}{}_{\min},\;approach_{\min}\right]=\left[4,2\right]$; (**b**) network $\left[s^{\left\{1\right\}}{}_{\min},\;approach_{\min}\right]=\left[6,6\right]$; (**c**) network $\left[s^{\left\{1\right\}}{}_{\min},\;approach_{\min}\right]=\left[11,4\right]$; and (**d**) network $\left[s^{\left\{1\right\}}{}_{\mathrm{best}},\;approach_{\mathrm{best}}\right]=\left[48,10\right]$.

After the graphical presentation of the performance function, regression and accuracy of particular models, it is now time to present the prognosis capability of the chosen networks. At first, Figure 17 shows the regression for the verification of an external specimen. There are included values of the Pearson coefficient at the top of each graph and equations for the linear regression line at the left side of each graph. One can observe that for the networks with 4, 6 and 11 neurons in the hidden layer the correlation between outputs and targets is on a very good level R≥0.997. On the other hand, a lack of correlation for the network with 48 neurons confirms considerable overfitting in this case (R=0.5992).

**Figure 18:**
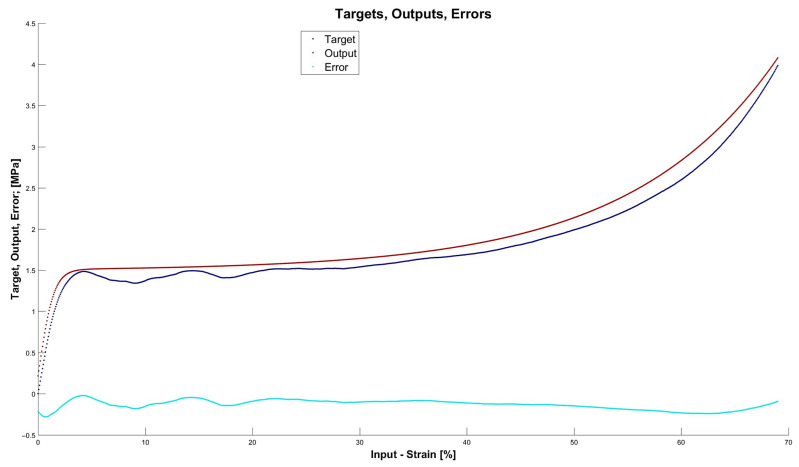
Prognosis of model $\left[s^{\left\{1\right\}}{}_{\min},\;approach_{\min}\right]=\left[4,2\right]$ (red dots). Additionally, errors (light blue) and targets (dark blue) are given.

**Figure 19:**
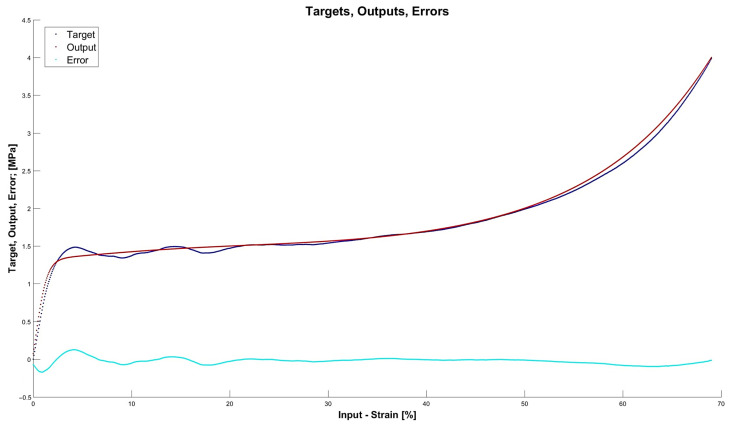
Prognosis of model $\left[s^{\left\{1\right\}}{}_{\min},\;approach_{\min}\right]=\left[6,6\right]$ (red dots). Additionally, errors (light blue) and targets (dark blue) are given.

**Figure 20:**
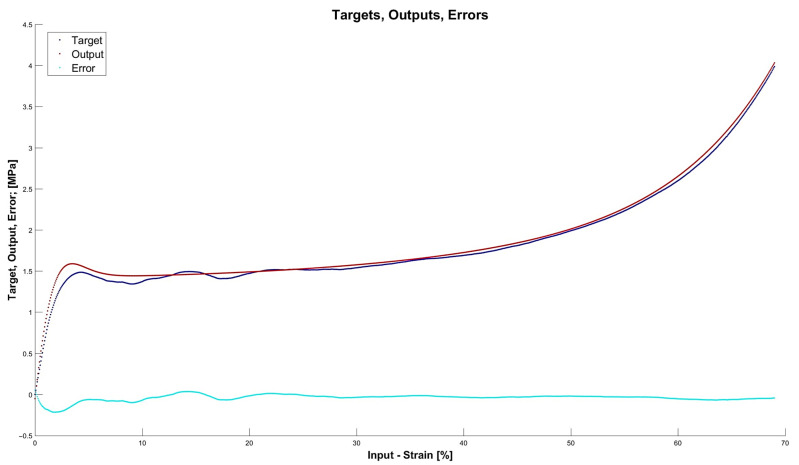
Prognosis of model $\left[s^{\left\{1\right\}}{}_{\min},\;approach_{\min}\right]=\left[11,4\right]$ (red dots). Additionally, errors (light blue) and targets (dark blue) are given.

**Figure 21:**
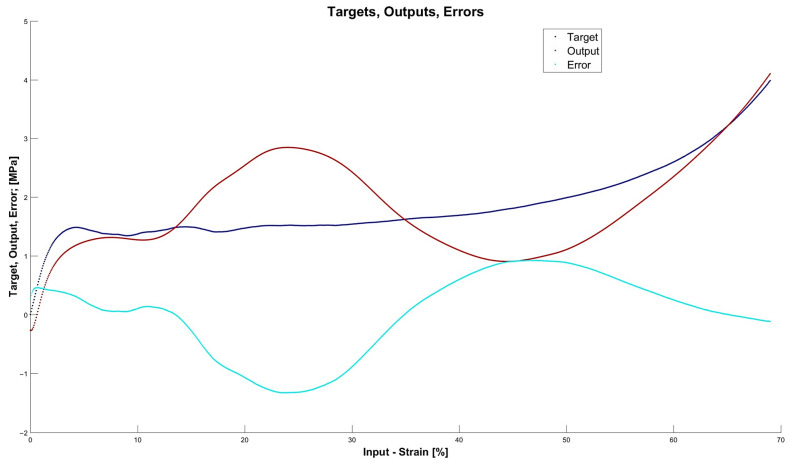
Prognosis of model $\left[s^{\left\{1\right\}}{}_{\min},\;approach_{\min}\right]=\left[48,10\right]$ (red dots). Additionally, errors (light blue) and targets (dark blue) are given.

Lastly, Figure 18, Figure 19, Figure 20 and Figure 21 present the detailed results of the prognosis of the chosen four models (red dots), errors (light blue) and targets (dark blue). In Figure 18 (network [4,2]) the prognosis is over the actual stress–strain plot. Figure 19 and Figure 20 show that for 6 and 11 neurons in the hidden layer large regions of the actual stress–strain plot overlap with the prognosed outputs. Figure 20 depicts how inaccurate the prognosis from the network with 48 neurons in the hidden layer is. Such a result is a consequence of the considerable overfitting in this network.

## 5. Conclusions

The presented research aimed to verify the possibility of describing the phenomenon of the compression of closed-cell aluminium by means of neural networks. Additionally, it was expected that specifications for a good-quality model would be found.

The starting point was the assumption of the general relationship between strength measures and apparent density for cellular materials: σ=f(ε,ρ) (2). Data from compression experiments were used to train neural networks varying in structure. The verification of the obtained models was a two-step procedure: 1. Verification of particular models built using an 11-sample data set was achieved, and 2. completely new data were introduced to the networks, and verification of prognosis was performed. A series of criteria (16)–(26) were proposed and used for the evaluation of accuracy, over- and underfitting and robustness. The study was performed in the following domains: strain ε∈〈0,69〉% and apparent density ρ∈〈0.200,0.297〉 g/cm^3^; furthermore, the specimen used for the second-step evaluation had an apparent density ρ=0.236 g/cm^3^.

Obtained results prove the hypothesis that neural networks are appropriate tools for building models of the phenomenon of the compression of aluminium foams. Additionally, the results enabled the identification of specifications of computations with artificial intelligence, which allows one to build good-quality models. These two general conclusions are now described in detail to list the specific contributions of our research:

1. The following neural network architecture specifications can be successfully used to model the addressed phenomenon: a two-layer feedforward NN with one hidden layer and one output layer. As for the activation functions, one may use the hyperbolic tangent sigmoid function in the hidden layer and the linear activation function for the output layer. As for the training algorithm, the Levenberg–Marquardt procedure was verified positively. For this procedure, the mean square error was used as the performance function with 0 as its goal. The learning rate and momentum should be calibrated; however, for the given experimental data and the number of neurons in the hidden layer assumed as 12 (near optimum) the results show that the influence of these two parameters was not the deciding factor. Values for momentum, learning rate, number of epochs to train, gradient and maximum validation failures, which were applied and recommended, are given in Table 2.
2. Regarding the number of neurons in the hidden layer, the interval $s^{\left\{1\right\}}=\langle 1;50\rangle$ was investigated. It was shown that even a relatively low complexity of four neurons can provide a satisfactory particular model and acceptable accuracy for the prognosis (${\left(MARE_{\mathrm{Test}}\right)}_{4,2}=4.5\%$, ${\left(MARE_{\mathrm{Verif}}\right)}_{4,2}=8.7\%$); nevertheless, the probability of obtaining such results by the first approach of training a model is low. Increasing the complexity by two neurons—up to six—considerably improves the accuracy of a particular model itself and prognosis (${\left(MARE_{\mathrm{Test}}\right)}_{6,6}=3.6\%$, ${\left(MARE_{\mathrm{Verif}}\right)}_{6,6}=2.7\%$); however, robustness is not satisfied for such networks. If one is interested in complying with insensitivity in the random assumption of weights and biases, networks with 11 neurons in the hidden layer provide robustness with a probability of 0.8 and a very good accuracy level at the same time (${\left(MARE_{\mathrm{Test}}\right)}_{11,4}=2.0\%$, ${\left(MARE_{\mathrm{Verif}}\right)}_{11,4}=3.0\%$). A greater number of neurons in the hidden layer (>11) also gives accurate results, but the accuracy is not increased substantially, and the overfitting risk is higher with 13 neurons or more.
3. In order to choose the model which most appropriately prognoses the mechanical characteristics of the studied materials, it is necessary to consider certain statistical measures for the assessment of the obtained results. In particular, evaluation parameters which indicate the occurrence of single instants of significant deviations between a mapped value and the respective target (e.g., *MaxARE*) should be introduced. Such individual considerable errors might disqualify a given model even if overall mean error would be on satisfactory level (for example *MARE*, *MSE*).
4. A series of criteria (16)–(26) is proposed to evaluate obtained models in a two-step evaluation. The idea of the two-step verification allows one to assess the fitting of the particular model to the data with which it was trained and to assess whether this particular model is capable of prognosing. Based on the presented research, it is recommended that the two-step model evaluation is performed with regard to the following qualities and measures explained in Section 3.5: accuracy ($MARE_{\mathrm{Test}}$, $MARE_{\mathrm{Verif}}$), under- and overfitting ($MARE_{\mathrm{Test}}$, $MARE_{\mathrm{Verif}}$, $\mathrm{av}\_MARE_{\mathrm{Test}}$, $\mathrm{av}\_MARE_{\mathrm{Verif}}$) and robustness ($SAE_{\mathrm{Valid}}$).
5. The relationship between the number of neurons in the hidden layer and convergence (meant as nearing to $MSE_{\mathrm{Valid}}=0$) can be very well described by a power law, which proves that the modelling of closed-cell aluminium during compression is not a chaotic but ordered phenomenon. However, at the same time the results show that for networks with 13 neurons and more, instances burdened with considerable overfitting start to occur. These two facts may indicate that in the pursuit of better accuracy, instead of increasing the number of neurons in the hidden layer {1}, one may choose to lower it while also adding another hidden layer. However, the multilayer network approach was beyond the scope of the presented work and is planned as further research.
6. None of the analyzed particular models had an accuracy in prognosis better than $MARE_{\mathrm{Verif}.\min}=1.661\%$. This threshold, below which even the most complex networks were unable to perform, is the premise for the idea that when using the tool of artificial intelligence, one has to balance the satisfactory demand of accuracy, network complexity and number of experimental data used for model training. The more data that are obtained from experiments, the better the accuracy, but the larger the computational time and costs of data harvesting also. On the other hand, if one agrees on some inevitable threshold of prognosis quality, they may be still be successful, but this still requires less time and cost investment.

As for the potential for further development, the following ideas seem interesting. One could assume another form of the initial relation (2), for example, by also incorporating morphological data of the material (e.g., cell wall thickness, average cell size) into the equation. Additionally, in the present research the verification of external data was performed for the specimen from the middle of the interval of density. One could extend this procedure to cross-validation and see how models would be capable of extrapolation. Other approaches could include using multilayer perceptions or different quality assessment criteria. Finally, one could investigate how neural networks model certain characteristics of closed-cell aluminium, which are important from material design or application engineering points of view—such research has already been started by the authors, the results of which are promising and will be published soon [95].

## Figures and Tables

**Table 1:** Characteristics of the samples.

Sample ID	*V* (mm^3^)	*m* (g)	*ρ* (g/cm^3^)
X_Z_02	122,902.09	36.55	0.297
Z_01	127,739.18	35.56	0.278
Z_02	126,160.72	35.90	0.285
Z_03	122,854.76	28.27	0.230
Z_05	124,804.39	26.72	0.214
X_Z_01_p	120,565.13	26.11	0.217
X_Z_06_p	110,950.83	24.83	0.224
X_Z_08_p	113,904.18	27.92	0.245
Z_06_p	125,270.04	28.13	0.225
Z_09_p	125,154.28	29.15	0.233
Z_12_p	122,038.14	24.36	0.200
Z_14_p	124,430.57	29.35	0.236

**Table 2:** Learning parameters for each *approach*.

Learning Parameter	Value
performance function goal	0
minimum performance gradient	10^−10^
maximum number of epochs to train	100,000
maximum validation failures	12
maximum time to train in seconds	infinity
learning rate	0.50
momentum	2.0

**Table 3:** Criterial measures and results from evaluation with criteria for accuracy (16) and overfitting (18).

$Crit1_{1}$	$s^{\left\{1\right\}}{}_{\mathrm{best}}$	$\;approach_{\mathrm{best}}$	$Crit1_{2}$
0.507%	48	10	35.049%

**Table 4:** Criterial measures and results from evaluation with Criteria (21) and (22).

$Crit2_{1.\mathrm{threshold}}$	$Crit2_{1}$	$s^{\left\{1\right\}}{}_{\min}$	$\;approach_{\min}$	$Crit2_{2}$	$\frac{\left|Crit2_{1}-Crit2_{2}\right|}{Crit2_{1.\mathrm{threshold}}}$
5%	4.455%	4	2	8.688%	85%
4%	3.572%	6	6	2.689%	22%
3%	2.767%	7	3	4.731%	65%
2.5%	2.313%	8	2	3.881%	63%
2%	1.959%	11	4	2.976%	51%
1.5%	1.497%	17	4	2.521%	68%
1%	0.997%	24	8	4.187%	319%

**Table 5:** Number of neurons in the hidden layer for which thresholds in Criteria (21) and (22) are fulfilled with av_MARE as the criterion measure.

$Crit2_{1.\mathrm{threshold}}$	$s^{\left\{1\right\}}{}_{\min.av.M.T.}$	$Crit2_{1}$	$Crit2_{2.\mathrm{threshold}}$	$s^{\left\{1\right\}}{}_{\min.av.M.V.}$	$Crit2_{2}$
5%	5	4.775%	10%	4	9.701%
4%	8	3.850%	9%	5	8.579%
3%	11	2.419%	8%	6	7.106%
2.5%	11	2.419%	7%	8	6.014%
2%	15	1.771%	6%	9	5.519%
1.5%	21	1.406%	5%	11	4.051%
1%	33	0.967%	4%	---	---

**Table 6:** Performance of chosen networks.

$\left[s^{\left\{1\right\}},approach\right]$	$MSE_{\min}$	$\frac{MSE_{\min}}{MSE_{\min,\left[4,2\right]}}$	epoch	$\frac{epoch}{epoch_{\left[4,2\right]}}$
[4,2]	0.0096888	1.00	1139	1.00
[6,6]	0.005076	0.52	1882	1.65
[11,4]	0.0016933	0.17	2539	2.23
[48,10]	0.00010296	0.01	5959	5.23

**Figure A1:**
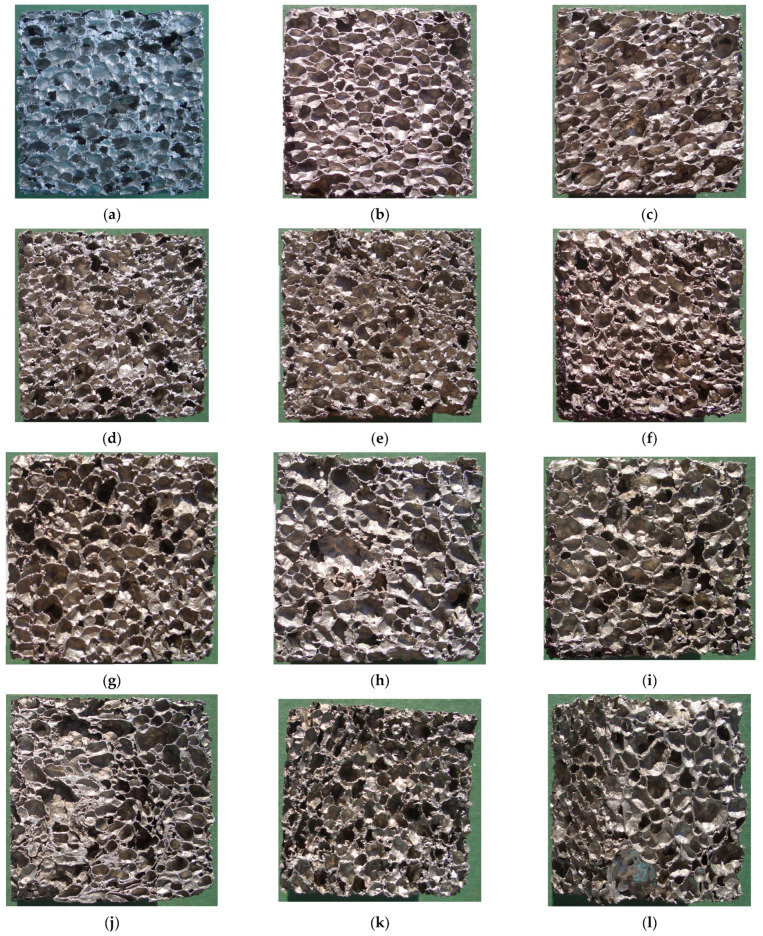
Specimens of metal foam: (**a**) Z_01, (**b**) Z_02, (**c**) Z_03, (**d**) Z_05, (**e**) Z_06_p, (**f**) Z_09_p, (**g**) Z_12_p, (**h**) Z_14_p, (**i**) X_Z_01_p, (**j**) X_Z_02, (**k**) X_Z_06_p, and (**l**) X_Z_08_p.

## Appendix B

The examples in Figure A2 demonstrate the effect of the parameter *p* used to smooth the stress–strain experimental data on the resulting NN-input dataset, as described in Section 3.1.1.

## Appendix C

In the calibration of learning parameters, which was described in Section 3.4, the momentum and learning rate were examined in relation to the performance function MSE. Here an additional remark is supplied to this analysis: the vast majority of the results falls into the interval $MSE_{\mathrm{majority}}\in \;\langle 0.0015;0.002\rangle \;{\mathrm{MPa}}^{2}$, and the maximum instance is not greater by one order. This may be interpreted as the negligible influence of the choice of learning rate and momentum on the NN training process within the investigated ranges.

## Appendix D

Figure 10 from Section 4 was scaled in order to present results clearly and legibly. Due to this graphic processing, some of the results could not fit in the drawing. The omission of some results in the plot in the main body of the article did not affect the conclusions or reasoning. Here the plot respective to Figure 10 from Section 4 is presented, but with all obtained results for the reader to have the complete picture—Figure A4.

## Appendix E

Here are provided figures that are complementary to Figure 12 from Section 4.3. Figure A5, Figure A6, Figure A7 and Figure A8 present plots of regression for all stages of network training (training, validation, test) and for the three stages cumulatively for the networks $\left[s^{\left\{1\right\}}{}_{\min},\;approach_{\min}\right]=\left[4,2\right]$, $\left[s^{\left\{1\right\}}{}_{\min},\;approach_{\min}\right]=\left[6,6\right]$, $\left[s^{\left\{1\right\}}{}_{\min},\;approach_{\min}\right]=\left[11,4\right]$ and $\left[s^{\left\{1\right\}}{}_{\mathrm{best}},\;approach_{\mathrm{best}}\right]=\left[48,10\right]$. Values of the Pearson coefficient are shown at the top of each graph. The equation for the linear regression line is given on the left side of each graph.
